# Recently recycled synaptic vesicles use multi-cytoskeletal transport and differential presynaptic capture probability to establish a retrograde net flux during ISVE in central neurons

**DOI:** 10.3389/fcell.2023.1286915

**Published:** 2023-11-06

**Authors:** Mason Parkes, Nathan L. Landers, Michael W. Gramlich

**Affiliations:** Department of Physics, Auburn University, Auburn, AL, United States

**Keywords:** synaptic vesicle, microtubule, axonal traffic, presynapse, hippocampus, actin

## Abstract

Presynapses locally recycle synaptic vesicles to efficiently communicate information. During use and recycling, proteins on the surface of synaptic vesicles break down and become less efficient. In order to maintain efficient presynaptic function and accommodate protein breakdown, new proteins are regularly produced in the soma and trafficked to presynaptic locations where they replace older protein-carrying vesicles. Maintaining a balance of new proteins and older proteins is thus essential for presynaptic maintenance and plasticity. While protein production and turnover have been extensively studied, it is still unclear how older synaptic vesicles are trafficked back to the soma for recycling in order to maintain balance. In the present study, we use a combination of fluorescence microscopy, hippocampal cell cultures, and computational analyses to determine the mechanisms that mediate older synaptic vesicle trafficking back to the soma. We show that synaptic vesicles, which have recently undergone exocytosis, can differentially utilize either the microtubule or the actin cytoskeleton networks. We show that axonally trafficked vesicles traveling with higher speeds utilize the microtubule network and are less likely to be captured by presynapses, while slower vesicles utilize the actin network and are more likely to be captured by presynapses. We also show that retrograde-driven vesicles are less likely to be captured by a neighboring presynapse than anterograde-driven vesicles. We show that the loss of synaptic vesicle with bound molecular motor myosin V is the mechanism that differentiates whether vesicles will utilize the microtubule or actin networks. Finally, we present a theoretical framework of how our experimentally observed retrograde vesicle trafficking bias maintains the balance with previously observed rates of new vesicle trafficking from the soma.

## 1 Introduction

Cytoskeleton-based trafficking mechanics have long been explored because of their essential role in neuronal function and maintenance (Westrum et al., 1983; Okada et al., 1995; Sorra et al., 2006; Perlson and Holzbaur, 2007; Tao-Cheng, 2007; Hirokawa et al., 2009; Staras and Branco, 2010; Tang et al., 2013; Wu et al., 2013; Maeder et al., 2014; Guedes-Dias et al., 2019; Gramlich et al., 2021; Watson et al., 2023). Protein trafficking via cytoskeleton transport is essential for synaptogenesis (Perlson and Holzbaur, 2007; Santos et al., 2009; Klassen et al., 2010; Wu et al., 2013; Guedes-Dias et al., 2019; Guedes-Dias and Holzbaur, 2019; Kurshan and Shen, 2019; Watson et al., 2023) and to replace older proteins with newer proteins for efficient function (Cohen et al., 2013; Dörrbaum et al., 2018, 2020; Heo et al., 2018; Truckenbrodt et al., 2018; Jähne et al., 2021; Watson et al., 2023). Protein recycling involves two steps: first, newly synthesized proteins from the soma are trafficked to presynaptic sites; second, older synaptic vesicles (SVs) must return to the soma from presynaptic sites.

Vesicles carrying newly synthesized protein, called synaptic vesicle precursors (SVPs), have been extensively studied and found to utilize microtubule (MT)-based transport to selectively traffic to presynaptic sites (Maeder et al., 2014; Yogev et al., 2016; Guedes-Dias et al., 2019) via MT-end sites (Guedes-Dias et al., 2019). What has been less understood but is of equal importance to the recruitment of new proteins is the trafficking mechanics of older vesicles that must transport aged proteins back to the cell soma for recycling. Older proteins must be replaced with new proteins at the same rate in order to maintain balance during neuronal maintenance and be dynamically modulated to accommodate presynaptic plasticity; otherwise, a buildup occurs, limiting efficient neuronal function. Recent studies show that proteins are regularly replaced depending on use and age (Cohen et al., 2013; Fornasiero et al., 2018; Truckenbrodt et al., 2018; Jähne et al., 2021). The average age of all presynaptic proteins measured *in vivo* is ∼10 days (Fornasiero et al., 2018); the average age reduces dramatically to 1–2 days for different neuron types as well as for active-zone- and exocytosis-related proteins that are used more frequently (Cohen et al., 2013; Fornasiero et al., 2018). However, one important missing piece from these aggregate protein turnover studies is how individual vesicle trafficking mechanics affect the rate at which these proteins are returned to the soma for recycling. Thus, the mechanics of older-vesicle trafficking are important to better understand how efficient neuronal function is maintained.

Despite extensive exploration, the mechanics of how older vesicles are trafficked back to the soma are not completely understood yet. We and others have shown that SVs that traffic older proteins engage in axonal transport through a process called inter-synaptic vesicle exchange (ISVE) (Park et al., 2012; Lee et al., 2012; Rizzoli, 2014; Joensuu et al., 2016; Gramlich and Klyachko, 2017; Chenouard et al., 2020; Chen et al., 2021; Park et al., 2022). We have shown that inhibiting actin-based transport during ISVE does not completely inhibit transport (Gramlich and Klyachko, 2017), but other studies have shown that SVs pause at the ends of actin filaments during ISVE even in the absence of MTs (Chenouard et al., 2020), suggesting that actin alone is essential for trafficking. These inconsistencies leave the question of whether ISVE involves MT-based transport unanswered. Establishing the discrepancy between MT- and actin-based transport is essential because actin filaments along the axon are randomly directed (Ganguly et al., 2015; Chenouard et al., 2020) and thus would not create a net flux toward the soma as expected to maintain the balance of proteins essential for neuronal function, whereas MT-based transport has been shown to support differential transport mechanics based on the difference in kinesin/dynein-driven transport (Yogev et al., 2016; Gramlich et al., 2021). This discrepancy leads to the following central goals of the present study: 1) to determine whether SVs utilize MT-mediated transport as well as actin-mediated transport and 2) to determine whether older SVs exhibit a net flux toward the soma.

We distinguish the mechanics of ISVE trafficking utilizing our previously established approach to label single SVs that have recently undergone exocytosis, along with an established approach to label MT ends. We then utilize a combination of advanced computational algorithms to quantify SV transport mechanics relative to MT-end locations. We show that SVs pause at the observed MT-end locations, consistent with previous observations of other SVPs. We then show that SV capture by presynapses during ISVE is speed-dependent and differentially regulated, with retrograde capture lower than anterograde capture. We show that the loss of myosin V is the dominant mechanistic pathway that determines whether SVs are trafficked locally or back to the soma. Finally, we develop a model of observed experimental transport and capture mechanics to show how older SVs have a net flux toward the soma in balance with previously observed SVP net flux from the soma. Our approach shows how motor motility mechanics are differentially regulated at the single-SV level in order to regulate SV turnover. Furthermore, our experimental parameter measurements combined with our computational model approach show how differentially trafficked SVs maintain a flux balance at the soma and protein clearance rate as a function of the distance from the soma.

## 2 Results

### 2.1 Synaptic vesicles use microtubules during axonal inter-synaptic vesicle exchange

**FIGURE 1:**
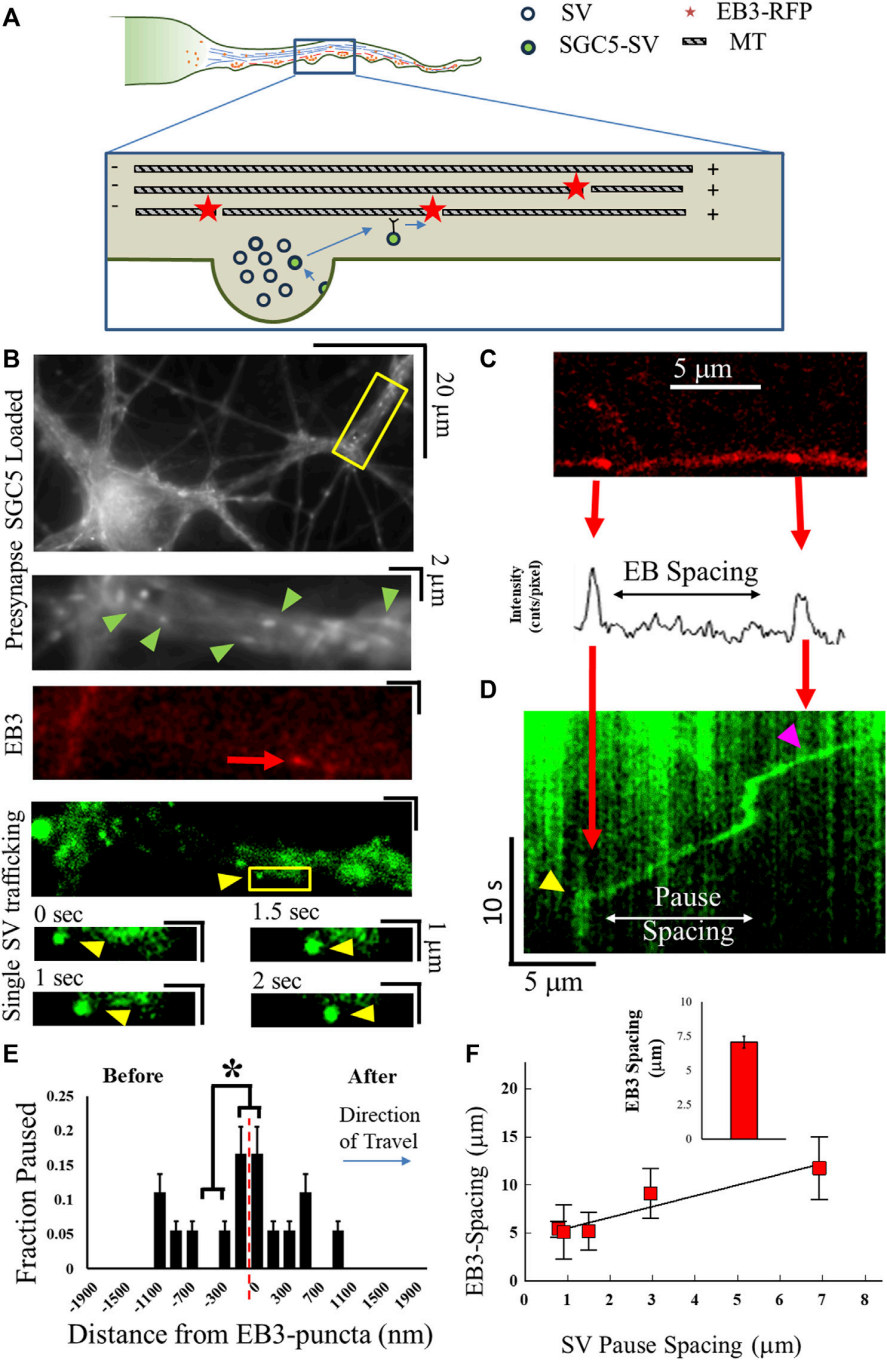
MT structure correlates with SV mechanics. **(A)** Cartoon of MT-supported SV motility along the axon. MT plus-end locations are labeled with EB3–RFP shown by red dots. SVs travel until reaching an MT end and pause before continuing motion. **(B)** Cultured cell images showing experimentally measured co-localization of labeled presynapses (green arrows), EB3–GFP puncta (red arrow), and single SGC5-labeled SVs (yellow arrows). Single SVs travel toward EB3-puncta locations (lower images). **(C)** EB3–RFP puncta are first imaged along axonal processes and quantified via fits to line-intensity analysis (lower panel). **(D)** Single SVs are then imaged, and kymograph analysis is used to identify locations of pausing (yellow arrows) or locations of traversal of EB3 puncta (magenta). **(E)** Co-localization of SV pausing with an EB3-punctum location is determined by quantifying the number of SV pauses as a function of distance from an EB3-punctum position (x = 0). SV pausing is separated before (−x axis) and after (+*x*-axis) it reaches the EB3 location. **(F)** SV-pause spacing correlates with local EB3 punctum spacing. The average EB3 punctum spacing (inset) is consistent with previously measured MT-end spacing. Single-pause events N_cultures_ = 2; N_samples_ = 3; N_SVs_ = 21. * = *p* < 0.1 from comparing two-proportion tests. SV-pause spacing correlation N_cultures_ = 2; N_samples_ = 6; N_SVs_ = 13. Fit to line *R* ^2^ = 0.97.

To establish whether SVs utilize MT transport during ISVE, we utilized our previously established approaches (see Methods) to correlate vesicle trafficking with the MT cytoskeleton (Gramlich et al., 2021). Many vesicles that are trafficked on the MT cytoskeleton are observed to pause at MT ends (Yogev et al., 2016; Guedes-Dias et al., 2019) due to motor-mediated mechanics (Gramlich et al., 2017; Gramlich et al., 2021; Guedes-Dias et al., 2019). Here, we measure recently recycled SVs that are trafficked along the axon and quantify the pause fraction as a function of position relative to labeled MT ends (Figure 1A). We distinguish MT plus-ends by labeling the end-binding protein-3 (EB3) commonly found at the dynamic plus-tip of MTs, using an EB3–RFP (Figure 1B) (Stepanova et al., 2003). We observed individual EB3 puncta with an average spacing of 7.07 μm (Figure 1F), consistent with previously measured MT-end spacing (Baas et al., 1988, 2016; Yu and Baas, 1994; Baas and Lin, 2011; Yogev et al., 2016).

We then observed that single SGC5-labeled SVs during ISVE traveled along the same location as the EB3 puncta (Figure 1D). SVs were observed to either pause at known EB3 locations (yellow triangles, Figure 1D) or traverse them (magenta triangle, Figure 1D). We then quantified that aggregate SVs pause as they approach or just pass EB3 puncta (Figure 1E) and observed that SVs preferentially pause with a combined probability of 32% ± 8% within 100 nm or 50% ± 11% within 500 nm.

We further correlated SV-pause spacing with locally observed EB3 spacing, regardless of whether SVs paused at EB3 puncta, and observed that SV-pause spacing increased with increasing EB3-puncta spacing (Figure 1F), with a measured correlation (*R* ^2^ = 0.97) between the EB3-puncta spacing and the spatial frequency of SV pauses. We note that the distance between SV pauses is approximately half the average distance between the measured EB3 points, which is consistent with previous observations stating that not all microtubule ends dynamically grow or shrink in neurons and would not have bound EB3 (Desai and Mitchison, 1997; Conde and Cáceres, 2009; Kapitein and Hoogenraad, 2015).

Taken together, these results suggest that recently recycled SVs utilize MT-based transport during ISVE. However, these analyses do not distinguish the directionality of SV mobility or whether SV pausing exclusively occurs at MT ends or whether actin dynamics are also involved.

#### 2.1.1 Synaptic vesicles exhibit unbiased retrograde mobility

**FIGURE 2:**
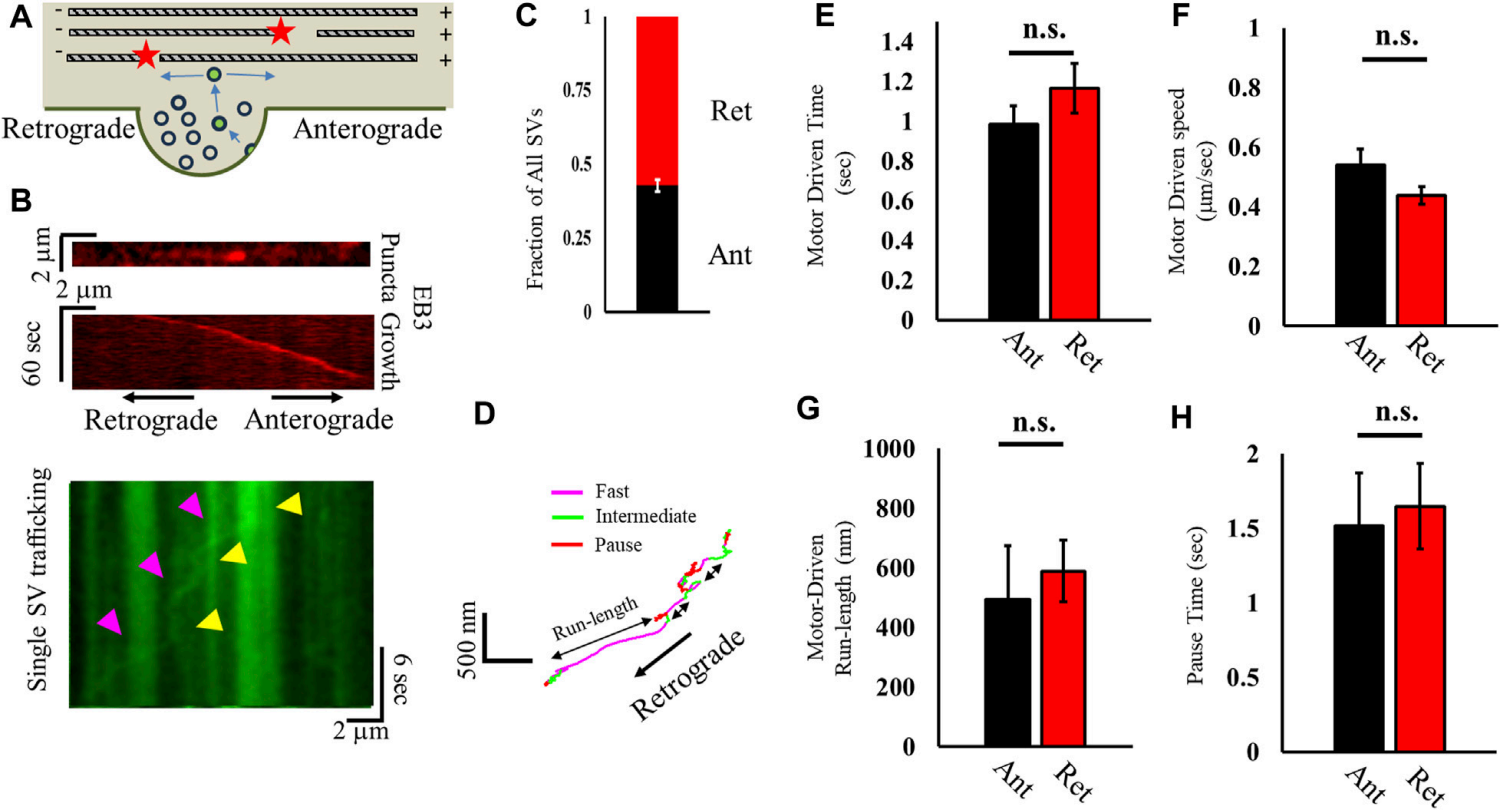
Correlation analysis distinguishes differences in anterograde and retrograde motor-driven motility. **(A)** Cartoon of recently recycled SVs leaving a presynapse and traveling either retrograde or anterograde. **(B)** Kymograph of slowly growing EB3 puncta (red) used to distinguish retrograde and anterograde motion (left). A kymograph of a single SV (green) imaged after EB3 was shown traveling in the opposite direction of the EB3 punctum growth, indicating retrograde motion. **(C)** Fraction of all SVs that travel in the retrograde (red) and anterograde (black) directions distinguished relative to EB3 punctum growth. **(D)** Bouts of motion (fast, intermediate, and pause) are differentiated using a correlation analysis approach, with a fast motion corresponding to motor-driven motility along a cytoskeleton. **(E–H)** Correlated motion metrics discern retrograde and anterograde motility for motor-driven speed **(E)**, time in motor-driven motion **(F)**, distance traveled during motor-driven motion **(G)**, and time spent in the paused state **(H)**. All SVs measured: N_cultures_ = 2; N_samples_ = 9; N_retrograde_ = 55; N_anterograde_ = 41. Retrograde motion: N_fast runs_ = 38; N_Pause-times_ = 44. Anterograde motion: N_fast runs_ = 61; N_Pause-times_ = 72. Statistics from a two-tailed KS test.

We next sought to determine whether ISVE SV mobility was directionally biased (Figure 2A) in order to distinguish whether a net bias mediates SV trafficking. Actin filaments are randomly oriented along the axon (Ganguly et al., 2015), and the processive motor myosin V can only travel in one direction along an actin filament (Baker et al., 2004); on the other hand, MTs are polarized along the axon (Figure 2A) and MT-based transport can occur bidirectionally, with kinesin-family motors mediating transport along the plus-end (Vale et al., 1996; Gennerich and Vale, 2009; Hirokawa et al., 2009) and dynein mediating minus-end transport (Gennerich and Vale, 2009; McKenney et al., 2014; Reck-Peterson et al., 2018). Furthermore, MT-based directional mobility can lead to a net flux because MT-based transport has a well-established differential bias in different mobility metrics, with kinesin speeds different from dynein speeds (Gennerich and Vale, 2009; Hendricks et al., 2010; Hancock, 2014).

To distinguish any potential directionality of SVs, we correlated SV mobility with EB3-punctum growth. We measured the slow directional motion of EB3 puncta (<0.25 μm/s; Figure 2B; Supplementary Figure S1) (Stepanova et al., 2003), which grow toward the anterograde direction, prior to imaging SV mobility. We then imaged SV mobility and correlated SV travel direction with EB3-punctum travel direction during ISVE (Figure 2B). The results revealed that recycled SVs traffic equally in both retrograde (57% ± 10%) and anterograde (43% ± 10%) directions during ISVE (Figure 2C).

To distinguish any bias in directional motor-driven mobility, we utilized our correlation analysis approach to determine whether bouts of motor-driven motility exhibited any directional bias (Figure 2D) (Gramlich and Klyachko, 2017). In brief, our approach distinguishes bouts of directed SV motion with a minimum speed (>0.2 μm/s), maximum angle of deviation during motion (<60°), and minimum time during motion (>0.5 s). We observed that the time spent (Figure 2E), speed (Figure 2F), and total run length (Figure 2G) during motor-driven mobility are independent of direction. We further observed that the pause time was independent of SV direction (Figure 2H).

Taken together, the combination of motility parameters suggests that SV trafficking is independent of direction, which would suggest support for the previously proposed actin-based motility since actin polymerization is unbiased (Gramlich and Klyachko, 2017; Chenouard et al., 2020). However, these results are also inconsistent with those showing that ISVE SV mobility is inhibited by MT ends (Figure 1) because MT-mediated cargo speeds have long been shown to be direction-dependent based on differences in motor speeds (Vale, 2003; Gennerich and Vale, 2009; Hendricks et al., 2010; Hancock, 2014; Reck-Peterson et al., 2018; Balseiro-Gómez et al., 2022). One possibility is that MT-mediated transport and actin-mediated transport are mixed together during analysis in order to obtain average results. Distinguishing actin and MT mechanics would thus require acutely changing one or the other cytoskeleton network and observing differences in mobility and pausing behavior, which we address in the following section.

#### 2.1.2 Synaptic vesicle ISVE and pause mechanics are both myosin V and microtubule dependent

**FIGURE 3:**
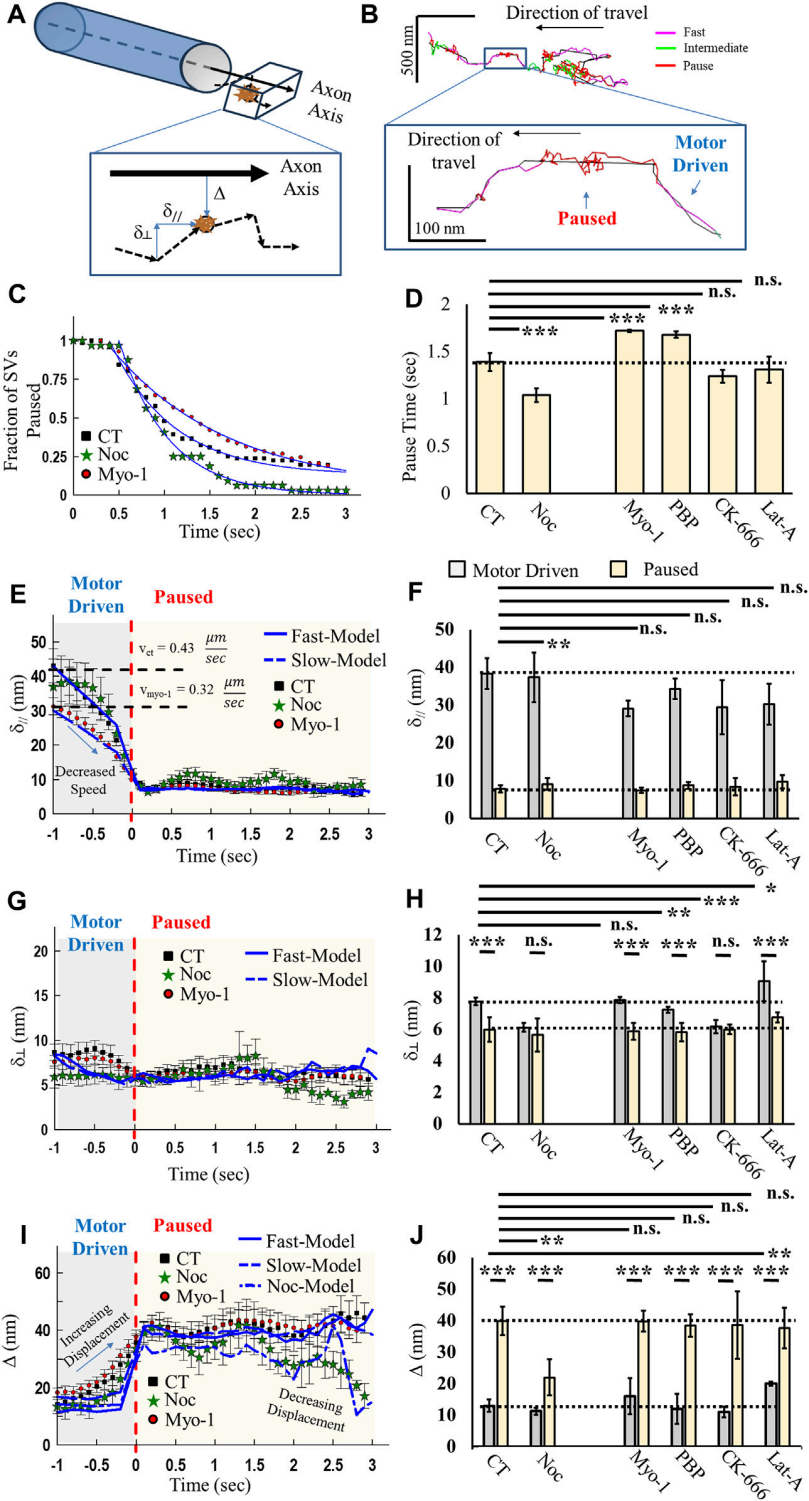
Vesicle mobility and pausing mechanics are microtubule- and actin cytoskeleton-dependent. **(A)** Model of axonal geometry analysis, based on SV track data, that distinguishes track motion parallel (δ_//_), perpendicular (δ_┴_), and displacement (Δ) relative to the axon central axis. **(B)** Example track color coded for correlated motion (fast, intermediate, and pause) with the identified central axonal axis (black line). Inset shows a zoomed-in section of track that shows fast mobility (magenta), followed by a pause (red). SV pausing shows motion away from the central axis, followed by a return to the central axis. **(C)** Aggregate SV time spent in the paused state for control (CT), myosinV-inhibited (Myo-1), and 2 nM nocodazole (Noc). **(D)** Mean fit pause times from exponential fits to data in **(C)**. **(E)** Aggregate SV parallel motion relative to the identified axon axis for control (black) and myosinV-inhibited (Myo-1). SV mobility is organized relative to the start of the pause (T = 0), with the identified fast motion (motor-driven), followed by paused motion. **(F)** Maximum SV parallel motion during motor-driven motion (gray) and during pausing (yellow). Statistical comparisons are carried out for parallel motion during a pausing condition. **(G)** Aggregate SV perpendicular motion relative to the identified axon axis. SV mobility is organized relative to the start of pause (T = 0), with the identified fast motion (motor-driven), followed by paused motion. **(H)** Maximum SV displacement during motor-driven motion (gray) and during pausing (yellow). **(I)** Aggregate SV displacement relative to the identified axon axis. SV mobility is organized relative to the start of the pause (T = 0), with the identified fast motion (motor-driven), followed by paused motion. **(J)** Baseline SV displacement during motor-driven motion (gray) and average SV displacement during pausing (yellow). CT: N_cultures_ = 9; N_samples_ = 18; N_SVs_ = 58; N_fast-runs/pause_ = 72. Myo-1: N_cultures_ = 9; N_samples_ = 12; N_SVs_ = 84; N_fast-runs/pause_ = 132. PBP: N_cultures_ =; N_samples_ =; N_SVs_ = 166; N_fast-runs/pause_ = 122. CK-666: N_cultures_ = 3; N_samples_ = 10; N_SVs_ = 46; N_fast-runs/pause_ = 12. Noc: N_cultures_ = 3; N_samples_ = 9; N_SVs_ = 56; N_fast-runs/pause_ = 32. Lat-A: N_cultures_ = 3; N_samples_ = 7; N_SVs_ = 71; N_fast-runs/pause_ = 43. * = *p* < 0.05, ** = *p* < 0.01, and *** = *p* < 0.001. Comparisons within data conditions (before/during pause) and comparisons across data conditions were obtained from the Mann–Whitney U-test.

To establish whether SVs utilize a combination of MT and actin transport during motor-driven and pausing mechanics, we extended our correlation analysis approach to quantify SV mobility before and during identified axonal pauses (Figure 3). We hypothesized that if SVs utilized a combination of MT-based and actin-based motility, then pausing mechanics would be sensitive to changes in either network. To test this hypothesis, we developed an advanced computational algorithm that utilizes high-resolution track positions to distinguish the geometry of the axon and then quantified SV displacement relative to the axon direction (Figure 3A). We distinguished the direction of the axon (axon axis, Figure 3A), assuming cylindrical geometry that has partial curvature in small segments (see Methods). We then quantified instantaneous motion relative to the axon axis as parallel (δ_//_, Figure 3A), perpendicular (δ_┴_, Figure 3A), or absolute displacement from the axon axis (Δ, Figure 3A). These metrics combined with correlation analysis identification of bouts of fast, intermediate, and pause motion allowed us to quantify the mechanics of SV mobility before and during a pause (Figure 3B). We identified locations of SV pausing (red, inset in Figure 3B) during axonal mobility that do not co-localize with the identified synapse locations.

To establish the role of MT-mediated and/or actin-mediated mechanics, we used an acute pharmacological approach to target different portions of MT or actin dynamics, and we selected concentrations of agents based on previously established concentration-dependent effects (see Methods). In brief, (i) to discern the role of MT-mediated transport, we exposed neurons to Nocodazole (Noc), which has been established to stabilize MT dynamics at nanomolar concentrations, resulting in paused MT dynamics (Vasquez et al., 1997); (ii) to determine the role of actin-mediated transport, we exposed neurons to micromolar concentrations of the myosin V-inhibiting agent MyoVin-1 (Myo-1) or nanomolar concentrations of pentabromopseudolin (PBP), which was previously shown to reduce myosin V-mediated motor-driven transport (Gramlich and Klyachko, 2017); and (iii) to determine the role of the actin structure, we exposed neurons to micromolar concentrations of latrunculin-A (Lat-A) or the ARP2/3-inhibiting agent CK-666 (CK-666), which destabilize polymerized actin or branched actin, respectively (Ganguly et al., 2015).

Time during SV pauses is differentially affected by MT- and actin-mediated motility. SV-pause time increases by ∼25% when myosin V is inhibited (Myo-1 and PBP, Figures 3C, D) compared to the control (CT, Figures 3C, D), whereas no difference was observed in pause time when actin is depolymerized or branched actin is inhibited (Lat-A or CK-666, Figure 3D). Alternatively, a significant decrease of ∼30% occurred in the time SVs paused when MTs are stabilized (Noc, Figures 3C, D). These pause-time results suggest that SV pauses represent a mixture of MT- and actin-mediated mechanics.

We next determined whether either MT- or actin-mediated mechanics affected SV motion in the direction of the axon axis before and during SV pausing. SV mobility parallel to the axon axis (δ_//_, Figure 3A) was high during periods of fast motion, consistent with motor-driven mobility along the cytoskeleton, but significantly reduced during pausing (Figure 3E). During fast SV motility, parallel SV mobility was reduced by 25%–30% in the absence of myosin V (Myo-1, Figure 3E) compared to the control (CT, Figure 3E), as previously shown (Gramlich and Klyachko, 2017). This approach also revealed that the time course of parallel motion shows that SV parallel mobility decreases prior to an SV-pause event at the same rate independent of Myo-1 (Figure 3E). Overall, motor-driven speed is not reduced in the presence of 1–2 nM nocodazole, and the same decrease in parallel SV mobility prior to pausing still occurs (Noc, Figure 3E). These results support our hypothesis that SV-trafficking mechanics are both actin- and MT-mediated.

To determine how SV pausing mechanics are MT-mediated, we quantified perpendicular SV motion, which exhibited similar mechanistic changes before/during pauses as observed in parallel motion (Figures 3G, H). Overall, perpendicular SV motion was lower during motor-driven motility than during parallel motion, as would be expected, but then decreased slightly during pausing (Figure 3F). However, perpendicular motion is also lower during motor-driven motion in the presence of either CK-666 or Noc than in both control samples and in the presence of Myo-1 (Figure 3G). Perpendicular SV motion remains constant during pausing except in the presence of Noc, where perpendicular motion begins to decrease 1.5 s after the start of the pause (Figures 3E, G).

Lastly, we compared SV displacement away from the axon axis to determine how far SVs travel during pausing. Displacement initially changes before/during pausing, supporting both parallel and perpendicular motion results. SVs begin to displace farther from the axon axis during motor-driven motion just prior to pausing (Figure 3I), consistent with a decrease in parallel motion during the same time period (Figure 3E). SVs undergo higher displacement from the axon axis during pausing under all conditions compared to motor-driven motion (Figure 3J). However, SV displacement quickly decreases with time during pausing in the presence of Noc (Figure 3I) and is significantly lower than in control samples (Figure 3J). This same decrease does not occur under any other condition (Figure 3J). These results suggest that previous measurements of single-SV motility have been made mixing both actin- and MT-mediated SV mobility and that focusing on one type of motion alone is insufficient to discern them.

To support our experimental results, we developed a computational model of SV mobility and pausing to distinguish how motility mechanics lead to measured experimental SV mobility parameters (see Methods). In brief, SVs were modeled using the following approach: (i) SVs travel along a fixed axis with a minimum speed and maximum angle of mobility; (ii) SVs begin to slow 10 time steps (1 s) prior to pausing; (iii) SVs then pause for a random amount of time; (iv) during pausing, SVs are spatially biased to a displacement distance (50–250 nm); and (v) SVs re-initiate fast motion until the end of the simulation. Simulated track positions are then run through the correlation algorithm and compared with experimental results (Figures 3C, E, G, I).

The simulated track results support experimental SV mobility mechanics in both space and time. During SV slowdown prior to pausing, SVs begin to decrease the forward speed along the axon and increase displacement away from the axon axis (solid blue lines, Figures 3E, I). In the absence of myosin V, forward motor-driven speed decreases and displacement away from the axon axis increases (dashed blue lines, Figures 3E, I). Furthermore, SVs are constrained to remain near the axon axis (Δ ∼ 45 nm, Figure 3I), but this is independent of myosin V mechanics (solid and dashed blue lines, Figure 3I). Instead, the time SVs spend in a paused state increases when myosin V is inhibited (solid blue lines, Figure 3C). These model results support the hypothesis that inhibiting myosin V alters the time until re-initiating motor-driven motility (Figure 3C). The nocodazole model results of displacement (dotted and dashed blue lines, Figure 3I) also support the EB3 results that SVs utilize the MT network. However, it is worth noting that our model/experiments are limited in that they cannot distinguish whether re-initiation of motor-driven motility is dominated by motor-binding kinetics alone or a combination of kinetic processes.

These experimental and computational results lead us to hypothesize two possible pathways of SV ISVE motility, namely, MT-mediated and actin-mediated transport:

(i) SVs utilize a combination of both MT- and actin-based motility, regardless of the direction, that would result in the observed unbiased average ISVE mobility. Furthermore, SVs can switch binding between MT and actin during pausing events.
(ii) SVs exclusively use either MT- or actin-based motility, with actin-based motility representing more local and frequently pausing SVs and MT-based motility representing faster and less frequent pausing.

These pathways can be distinguished when considering the known role of actin-mediated motility in SV capture by presynapses during ISVE (Darcy et al., 2006), which we will take advantage of next to discern which of the hypothesized pathways SVs utilize.

### 2.2 SVs exhibit direction-dependent and speed-dependent two-state trafficking mechanics near presynapses

To distinguish between the two aforementioned hypothesized mobility pathways, we quantified recycled SV capture by presynapses to determine whether the mobility of SVs that traverse presynapses is different from that of SVs captured by presynapses. It has been well established that actin is essential for vesicle capture (Darcy et al., 2006; Denker and Rizzoli, 2010) and for SV pool maintenance in order to maintain efficient presynaptic function (Cingolani and Goda, 2008; Bleckert et al., 2012; Miki et al., 2016). However, while MTs are observed at presynapse locations (Guedes-Dias et al., 2019) and are utilized for SVP targeting to presynaptic locations (Guedes-Dias et al., 2019), it is still unclear how MT transport mediates recycled SV motility at presynapses. Separately, even if recycled SVs do not exhibit a large retrograde bias in mobility during ISVE, differential capture mechanics by presynapses may correlate with SV direction. The scientific premise of the differential capture hypothesis is based on a previously reported net capture bias for SVPs during retrograde motion (Yogev et al., 2016; Balseiro-Gómez et al., 2022).

**FIGURE 4:**
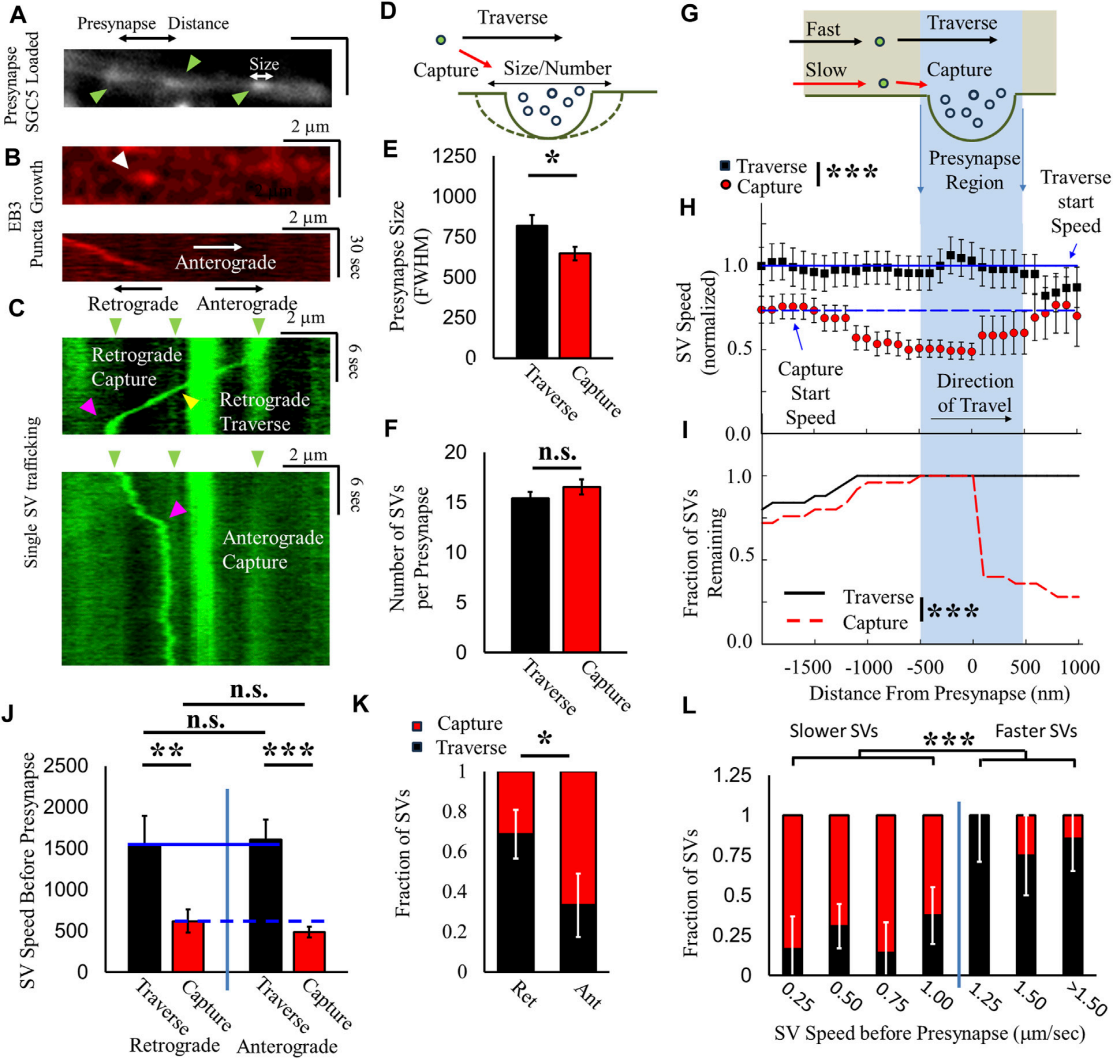
SV velocity and direction mediate presynaptic capture probability. **(A)** Presynapses are identified as bulk SGC5-loaded puncta (green arrows). **(B)** EB3–RFP (red kymograph) growth direction (white arrow) is identified relative to presynaptic puncta and corresponds to the anterograde axonal direction. **(C)** Single-SGC5 SVs (green kymograph) are recorded during ISVE transport near presynaptic puncta (green arrows). SGC5 is observed to either traverse (yellow arrows) or pause (magenta arrows) at presynaptic positions. Retrograde-driven SVs exhibit more traversal events than pauses at presynapses. Anterograde-driven SVs exhibit more pause events at presynapses than traverses. **(D)** Cartoon model hypothesizing whether SV capture/traverse by presynapses is dependent on presynapse size and SV recycling pool number. **(E)** SVs are separated as either traversing an identified presynapse (black) or captured by presynapses (red), and presynapse size [white arrow panel **(A)**] is defined as the FWHM size of the SGC5 intensity. **(F)** Number of SVs within presynapses, determined by the relative presynapse SGC5 intensity **(A)** divided by single-SGC5 intensity **(C)** for ISVE SVs that traverse (black) and are captured (red) by presynapses. **(G)** Cartoon model of hypothesized SV speed prior to presynapses is different for captured/traversed SVs. **(H)** Relative SV speed for SVs that are captured (red) is lower than that for SVs that traverse (black) presynapses. Aggregate SVs are compared to their starting speed for captured (blue dashed line) and traversed SVs (blue solid line). **(I)** Fraction of captured SVs (red dashed line) and traversed SVs (black solid line) as a function of distance from the presynapse. A small fraction of captured SVs remain within the presynapse and are then observed to leave the presynapse after capture. **(J)** SV speeds before reaching a presynapse are separated by direction (retrograde and anterograde). Traversing SV speed (black) is significantly larger than captured SV speed (Red) for retrograde and anterograde directions. Traversing (solid blue line) and captured SV speeds (dashed blue line) are the same for retrograde and anterograde directions. **(K)** The fraction of SVs that traverse presynapses (Black) is higher for retrograde motility (>70%) and lower for anterograde motility (<30%). **(L)** Capture/traverse fractions are separated by speed to determine the speed-dependent relationship. Slower-moving SVs (<1 μm/s) have a lower traverse fraction and higher capture fraction than faster-moving SVs (>1 μm/s). Directional capture/traverse: N_cultures_ = 3; N_samples_ = 9; N_retrograde_ = 19; N_anterograde_ = 15. Capture/Traverse: N_cultures_ = 4; N_samples_ = 5; N_SVs_ = 23 * = *p* < 0.1, ** = *p* < 0.01, and *** = *p* < 0.001. Fractional capture statistics obtained by comparing two-proportion tests. Distance-dependent speed and fraction comparisons under data conditions (speeds before/at the presynaptic region) were obtained from the repeated-measures pair-wise *t*-test, and comparisons under data conditions were obtained from the Mann–Whitney U-test. Presynapse size, number, and direction-dependent SV speeds statistics were obtained from the two-tailed *t*-test.

We correlated SV mobility, as mentioned previously (Figure 1), relative to presynapses that exhibit bulk loading of SGC5 (Figure 4A), using our previously established approach (see Methods) (Forte et al., 2017; Gramlich and Klyachko, 2017). We identified the axonal MT polarization direction by imaging EB3-punctum growth (Figure 4B). We then correlated single SVs undergoing ISVE near both the identified presynapses and EB3 puncta (Figure 4C). We then defined an SV traverse event as any SV that does not stop when traveling across an identified presynapse location (black arrow, Figure 4D). Conversely, we define a capture as any SV that remains within ±500 nm of a presynaptic geometric center longer than the time for diffusion across a presynapse (>3 s), which would indicate that the SV is constrained within the presynaptic recycling pool (red arrow, Figure 4D).

We first determined whether the properties of the presynapse mediated whether SVs will traverse or be captured by the presynapse by correlating the presynapse geometric size or number of SVs within the recycling pool (Figure 4D). Presynapses where SVs traverse are larger (∼25%) than presynapses where an SV is captured (Figure 4E). However, the number of SVs within the presynapse does not mediate whether an ISVE SV is captured by or traverses the presynapse (Figure 4F). These results suggest that the structure and organization of the presynapse influence the probability of an SV being captured or traversing a presynapse. However, they do not indicate whether the mediating factor is based on SV motility prior to the presynapse or if the presynapse structure is the dominant factor, which we address in the following paragraph.

We established whether SV speed is correlated with the fraction of SVs that were captured or traverse presynapses based on the following premise: if SVs utilize either the MT or actin cytoskeleton [aforementioned pathway (ii)] and capture mechanics are based exclusively on the actin network, as previously shown (Darcy et al., 2006), then SVs that traverse a presynapse would have different speeds from those of SVs that are captured by a presynapse, based on differences in molecular motor speeds (Figure 4G). Aggregate speeds for SVs that are captured at presynaptic locations are initially slower (<75%) 1 μm prior to capture (red, Figure 4H) compared to the speeds of SVs that traverse and travel faster (black, Figure 4H) 1 μm prior to presynaptic locations. Furthermore, captured SV speeds begin to decrease <1 μm before reaching a presynapse compared to their initial baseline (dashed line, Figure 4H). Alternatively, traversing SV speeds remain relatively constant throughout their travel (solid line, Figure 4H). We also observed that the majority of captured SVs do not continue to travel beyond the presynaptic region during the period of observations (<2 min), while every observed traversing SV continues to travel past the presynaptic region (Figure 4I). A minority of SVs that are captured by a presynapse (<25%, red dashed line, Figure 4I) continue past the presynaptic region, after an extended period of time within the presynapse, and return to their original speed (red circles, Figure 4I) but remain slower than traversing SVs. These results support the hypothesized pathway (ii) that SVs utilize either MT-based or actin-based motility but not both.

We next focused on whether SV direction of travel influenced speed and/or capture/traverse fraction. We hypothesize that if SVs utilize either MT or actin-mediated motility, then their traverse and capture speeds should be independent of direction because we did not observe a direction-dependent motor-driven motility during ISVE (Figure 2). Both anterograde and retrograde directions had the same speed-dependent difference between SVs that traverse or are captured by presynapses (Figure 4J). Furthermore, travel speeds for traversing SVs were equal in anterograde and retrograde directions, while travel speeds for captured SVs were also similar (Figure 4J). We then quantified SV capture/traverse fraction based on the direction of travel before reaching a presynapse, and SVs traveling in the retrograde direction were captured at the presynapse ∼30% ± 15% of the time, whereas SVs traveling in the anterograde direction before reaching a presynapse were captured 68% ± 12% of the time (Figure 4L). These combined directional results suggest that SV motility is independent of travel, but SV capture/traverse of presynaptic sites depends on the direction of travel.

Lastly, we sought to determine whether the SV capture/traverse fraction changed continuously as a function of SV speed or was discontinuous. If SVs follow our hypothesized pathway (ii) and use either MT- or actin-based motility and SV capture occurred predominantly by slow-traveling SVs, then the SV capture/traverse fraction should exhibit a discontinuous distribution; this discontinuous distribution should show that slow-moving SVs are predominantly captured, while fast-moving SVs traverse presynapses (Figure 4G). Combined SVs with speeds less than 1 μm/s were predominantly captured by presynapses (*slower SVs*: capture >75%, Figure 4L), while SVs with speeds greater than 1 μm/s predominantly traversed presynapses (*faster SVs*: traverse >75%, Figure 4L). Rather than exhibit a continuous change in the capture/traverse fraction with increasing speed, SVs exhibit a discontinuous shift. The discrete shift in the capture/traverse fraction results further supports the hypothesized pathway (ii) that SVs utilize either MT- or actin-based motility but not both.

The combined SV results of capture/traverse fraction, speed, and direction support a two-state transport model, where recently recycled SVs leaving a presynapse either utilize MT- or actin-based motility but not both. However, the results do not distinguish how SVs are selected to transport either on the MT or actin networks, which we address in the following section.

### 2.3 SV bound myosin V mediates two-state capture/traverse mechanics at presynapses

**FIGURE 5:**
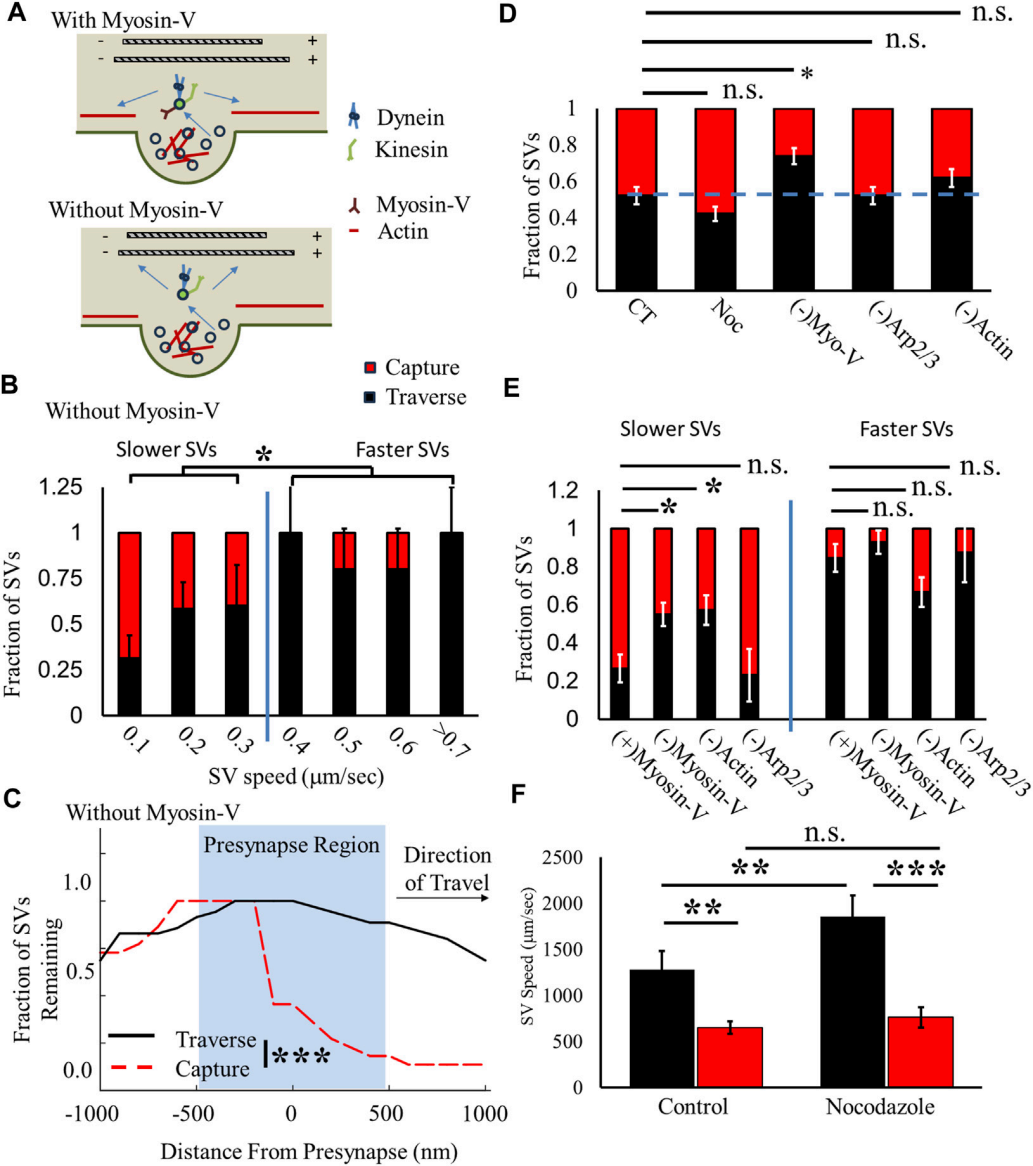
Myosin V/actin mediates slow-SV capture fraction but not fast-SV capture fraction. **(A)** Hypothesized model of two-state recycled SV ISVE transport. SVs with myosin V attached predominantly use actin cytoskeleton motility (top panel). SVs without myosin V attached utilize MT cytoskeleton motility (bottom panel). **(B)** The SV presynaptic capture fraction decreases with increasing SV speed similar to CT (Figure 4L). **(C)** The fraction of SVs undergoing ISVE without myosin V decreases for captured SVs but not for traversing SVs, similar to CT (Figure 4I). **(D)** The overall fraction of SVs that traverse presynapses increases without myosin V but does not significantly change under any other condition. **(E)** The traverse fraction for slower-moving SVs increases without myosin V [(-)Myosin V] or polymerized actin [(-)Actin], but traverse rates are unchanged for faster SVs. **(F)** Traversing SV speed (black) increases in the presence of 2 nM nocodazole, but captured SV speed (Red) is unchanged in the presence of nocodazole. CT: N_cultures_ = 4; N_samples_ = 5; N_SVs_ = 23. Noc: N_cultures_ = 3; N_samples_ = 6; N_SVs_ = 26. (-)MyosinV (combined Myo-1 and PBP conditions): N_cultures_ = 15; N_samples_ = 24; N_SVs_ = 68. (-)Arp2/3: N_cultures_ = 1; N_samples_ = 5; N_SVs_ = 21. (-)Actin: N_cultures_ = 3; N_samples_ = 7; N_SVs_ = 16. * = *p* < 0.1, ** = *p* < 0.01, and *** = *p* < 0.001. Fractional capture/traverse probability was obtained by comparing two-proportion tests. Comparison within data conditions (speeds before/at the presynaptic region) was obtained from the repeated-measures pair-wise *t*-test. Comparisons across data conditions were obtained from the Mann–Whitney U-test.

To discern the selectivity question, we utilize the quantified speed differences between traversing and captured SVs and known speed differences between MT-mediated and actin-mediated molecular motor motility. MT motor motility (dynein/kinesin) is consistent in the faster SVs that traverse presynapses (>1.0 μm/s, Figure 4L), (Vale et al., 1996; Hirokawa et al., 2009), whereas the actin-based molecular motor myosin V speed is consistent in slower SVs that are captured by presynapses (<1 μm/s) (Baker et al., 2004; Gramlich and Klyachko, 2017). We hypothesize that the loss of SVs with bound myosin V mediates whether recently recycled SVs that leave a presynapse will engage in MT- or actin-based motility (Figure 5A). If recently recycled SVs with bound myosin V leave a presynapse, then they travel along the polymerized actin network (top panel, Figure 5A). If SVs without myosin V leave a presynapse, then they travel long the MT network (bottom panel, Figure 5A). We test this hypothesis by acutely inhibiting myosin V, polymerized actin, or branched actin and measuring SV capture/traverse fraction and changes in speed.

We first quantified capture/traverse fractions as a function of SV speed to establish whether the loss of myosin V differentially affects the two-state capture/traverse fractions (Figure 4L). We hypothesized that if myosin V mediates slower SVs, then the loss of myosin V should reduce slow-SV capture fractions but not affect fast-SV capture fractions. The results of myosin V-inhibited SVs were combined with Myo-1 and PBP experiments as they resulted in the same changes (Figure 3; Supplementary Figure S2). First, SVs still exhibit a differential capture/traverse fraction in the absence of myosin V (Figure 5B). Slower SVs (<0.4 μm/s, >50% capture) had a higher SV capture fraction than faster SVs (>0.4 μm/s, <20% capture). It is worth noting that we previously showed that the loss of myosin V increases SV pausing, decreases SV run-length, and decreases measured SV speed for all SVs regardless of motility (Gramlich and Klyachko, 2017), which is why the overall measured SV speed is lower than that under control conditions (Figure 4L); however, the same two-state capture/traverse discontinuous change still occurs in the absence of myosin V. Furthermore, slower SVs in the absence of myosin V had a lower capture fraction than those under control conditions.

Importantly, the difference in the slower-SV capture/traverse fraction observed is not due to a change in the mechanics of where SVs are captured by presynapses. We quantified the fraction of SVs as a function of distance from presynapses, and captured SVs occur within the same presynaptic region (Figure 5C), as observed under control conditions (Figure 4I). Furthermore, the fraction of SVs that traverse presynapses did not significantly change within the presynaptic region (Figure 5C) but showed a faster reduction with distance than that under control conditions (Figure 4I). This difference is due to a reduction in the total SV travel distance, as previously shown (Gramlich and Klyachko, 2017). These results support our hypothesis that the loss of myosin V mediates slow-SV capture/traverse fraction but not fast-SV capture/traverse fraction.

To further support our hypothesis that myosin V mediates SV capture by presynapses, we compared the aggregate SV capture/traverse fraction under different MT and actin conditions regardless of the direction of travel (Figure 5D). We observed that half of all SVs were captured (48% ± 5%) under control conditions (CT, Figure 5D), and there was no significant change in capture fraction in the presence of 2 nM nocodazole (43% ± 14.2%). However, there was a significant decrease in SV capture fraction when myosin V was inhibited [(-)Myo-V, 26% ± 4%, Figure 5D] but not when ARP2/3 was inhibited [(-) Arp2/3 = 48% ± 5%, Figure 5D). Furthermore, loss of actin showed a decreased capture fraction [(-)Actin, 38% ± 5%, Figure 5D] but this was not significant (*p* = 0.2) due to the increase in SV capture by faster-moving SVs (described in the following paragraph). These results support the hypothesis that the loss of myosin V mediates the capture of SVs by presynapses.

To support our hypothesis that the loss of myosin V mediates the SV capture of slower SVs, we compared the SV capture/traverse fraction for slow and fast SVs under all acute conditions (Figure 5E). We separated all conditions into slower-moving SVs (SVs with a speed <50% of the maximum) and faster-moving SVs (SVs with a speed >50% of the maximum). The loss of myosin V [(-)Myosin V, 45% ± 6%, Figure 5E] and loss of actin [(-)Actin, 43% ± 8%, Figure 5E] both show a decrease in capture fraction for slower-moving SVs compared to the control [(+)Myosin V, 75% ± 7%, Figure 5E]. Alternatively, the loss of branched actin does not change the capture fraction for slower SVs [(-)Arp2/3, 76% ± 13%, Figure 5E]. In contrast, the loss of myosin V or ARP2/3 does not significantly change the capture fraction of faster SVs. The loss of actin increases the capture fraction of faster SVs compared to all other groups, but there were a low number of observed SVs traveling fast in the absence of actin.

We then compared the speed of SVs that traverse or are captured by presynapses in the presence of 2 nM nocodazole to distinguish whether acutely changing the MT network differentially affected faster or slower SVs. We hypothesize that if myosin V mediates slow-SV motility and capture and MT transport mediates fast-SV motility, then altering the MT network should only affect faster SVs. Speeds of SVs that traverse a presynapse (black, Figure 5F) are higher than those of SVs captured by presynapses (red, Figure 5F) for both the control and in the presence of nocodazole. However, the traverse SV speed significantly increases (46%) in the presence of 2 nM nocodazole, while the capture SV speed does not significantly change (∼15%). This result suggests that fast-SV motility is predominantly mediated by MT transport.

These combined capture/traverse results support our hypothesized two-state SV transport pathway (ii) that SVs exclusively utilize either MT- or actin-mediated mechanics. Furthermore, bound myosin V is the mechanism that mediates how SVs select the cytoskeleton network. SVs with bound myosin V utilize actin-mediated transport, while SVs without bound myosin V utilize MT-mediated transport.

### 2.4 Two-state SV ISVE trafficking provides the basis for the net bias of recycled SVs toward the soma

**FIGURE 6:**
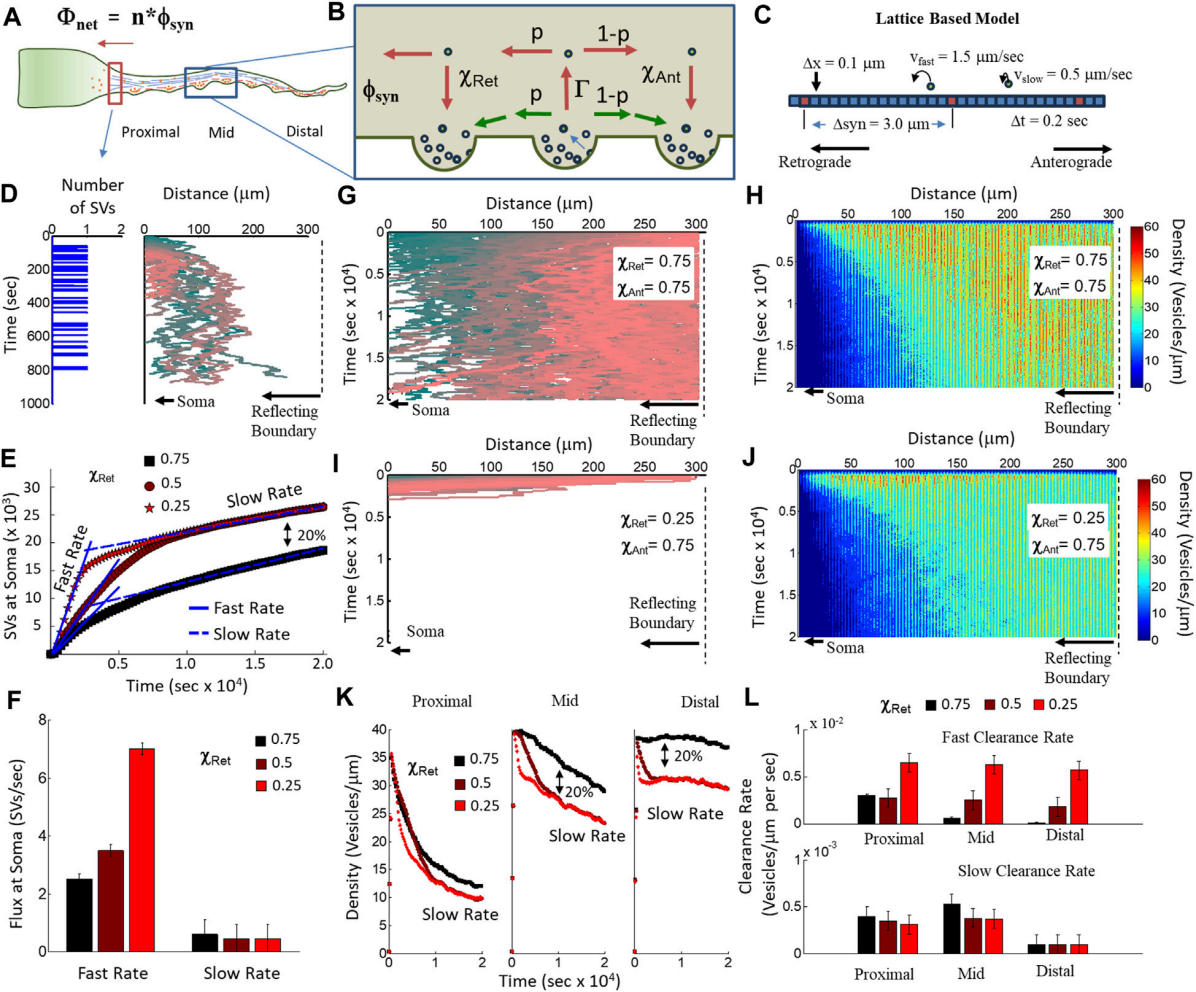
Computational model of two-state recycled SV motility and capture. **(A)** Net SV flux into the soma (Φ_net_) is the total number of presynapses (n) × the mean fractional flux per presynapse (ϕ_net_). **(B)** Two-state model of the fractional flux per synapse. A fraction of SVs that leave a presynapse engages in MT-mediated transport (Γ), have a probability of travel in the retrograde direction (p), and traverse a neighboring presynapse during ISVE (χ_ret_), creating a net flux toward the soma (ϕ_syn_). **(C)** Lattice-based model of axonal transport using the two-state model parameters. SVs hop along the axonal lattice at one of the two possible fixed velocities (slow and fast). Presynapses are separated by fixed distances (Δsyn). Each lattice site has a fixed length (0.1 μm), and each time step in the simulation is a fixed amount (0.2 s). **(D)** Example of simulated SV release from a single presynapse 10 μm from the soma. The presynapse releases a single SV every 6 s (right panel). SVs are captured by other presynapses along the axon with a fixed probability and random pause time. SVs that reach the soma are counted as captured (left panel). **(E)** Net flux of SVs at the soma as a function of time from simulations of 300 presynapses, each releasing an SV every 6 s for a total of 200 s (60,000 SVs). Each simulation has 80% slow-moving SVs and 20% fast-moving SVs. Fast-moving SVs have a retrograde capture probability of 0.75 (black squares), 0.5 (red circles), and 0.25 (red stars). Each simulation shows an initial fast flux rate and then a slow flux rate. **(F)** Fit flux rates for fast and slow rates in **(E)**. The fast flux rate increases with decreasing retrograde capture probability, representing the 20% fast-moving SVs. The slow flux rate is constant under all conditions, representing SVs with equal retrograde/anterograde capture probability (independent of speed). **(G)** Example of simulated tracks for a single SV released from each presynapse along the axon. All SVs have equal retrograde and anterograde capture probability (0.75). **(H)** Simulated spatial density of SVs per micron from a distribution of 200 SVs released from 300 presynapses. Each SV has an equal retrograde and anterograde capturer probability (0.75). **(I)** Example of simulated tracks for a single SV released from each presynapse along the axon. All SVs have a lower retrograde capture probability (0.25) than anterograde capture probability (0.75). **(J)** Simulated spatial density of SVs per micron from a distribution of 200 SVs released from 300 presynapses. 20% of SVs have a lower retrograde capture probability (0.25), while 80% of SVs have an equal retrograde and anterograde capturer probability (0.75). **(K)** Time-dependent change in SV density per micron along the axon for proximal (left panel), middle (middle panel), and distal (right panel) regions to the soma. **(L)** Quantified clearance rate of the number of SVs per micron per second is a measure for how quickly recycled SVs can be removed from a region along the axon. SVs proximal to the soma are cleared quickly regardless of the retrograde capture probability. A differential capture probability allows for SVs distal from the soma to be cleared faster than would occur for equal capture rates.

In this section, we propose an SV ISVE trafficking model that estimates the net flux of recycled SVs into the soma at the axon initial segment based on a mean-field approximation framework (Figure 6A) using our two-state transport model hypothesis (Figure 5) and our experimentally observed parameters supporting a net bias in recycled SVs toward retrograde motion (Figure 4). In this model, SVs either use slow-mediated motility with an equal capture probability in retrograde (χ_ret_, Figure 6B) and anterograde directions (χ_ant_, Figure 6B) or fast-mediated motility with a lower retrograde capture probability than anterograde. We then propose that each presynapse generates a steady-state net flux of SVs per second back to the soma (ϕ_syn_, Figure 6B). Each presynapse releases a recently recycled SV into the axon at a constant rate (ζ). A minor fraction of the released SVs travel with fast motility (Γ, Figure 6B), and a majority of SVs traffic with slower motility. Both fast and slow SVs have an equal probability of traveling in retrograde (p, Figure 6B) and anterograde directions (1-p, Figure 6B). These combined mechanisms are then repeated at each neighboring presynapse to result in a net flux per presynapse:

$$\phi_{syn}=\zeta *p*\Gamma *\chi_{Ret}.$$ (1)

We then propose that the total flux of recycled SVs into the soma (Φ_net_, Figure 6A) is equal to the number of SVs generated per presynapse (ϕ_syn_, Figure 6B) × the total number of presynapses along the axon:

$$\Phi_{net}=\sum_{i=1}^{n}\phi_{syn}.$$ (2)

We estimate the net flux per synapse from our observed results combined with our previously measured presynaptic release rates (Gramlich and Klyachko, 2017). We first estimate the rate at which each presynapse releases an SV at the fraction of released SVs per second (0.013 fraction/sec) × the average SV pool size of 40–80 SVs (Alabi and Tsien, 2012), which results in a steady rate of one SV every 0.5–1 s ( ζ*Pool Size ). We then estimate the fraction of SVs that traffic using fast transport as the fraction of SVs experimentally observed with a speed greater than 1.25 μm/s (Γ ∼ 20%–30%). Lastly, we estimate that the fraction of SVs trafficking to the soma can bypass neighboring presynapses (χ_Ret_ = 0.25) based on our observed retrograde capture/traverse fractions (Figure 4F).

These per-presynapse parameter estimates can then be combined with an estimate of the number of presynapses along an axon to obtain the steady-state net flux of recycled SVs. The combined per-presynapse rates result in an average of 0.015–0.03 SVs per second. If we estimate the number of presynapses along the axon to be between 300 and 500 for the cultured cell approach, then the combined net flux would result in between 4 and 15 SVs per second entering the soma. We note that this rate is consistent with a recently measured rate at which SVPs enter the axon carrying new proteins that are trafficked to presynapses to replace recycled SVs (Watson et al., 2023). Consequently, this model results in a net balance of vesicles within the axon.

To support this two-state model, we developed a computational model of recycled ISVE SV transport (see Methods). We chose a transient release approach to determine the total flux into the soma as well as measure how SVs are cleared from the axon as a function of the distance from the soma. In this approach, we modeled 300 presynapses along a one-dimensional lattice with their geometric centers separated by 3 μm, equal to the experimentally average presynapse distance (Gramlich and Klyachko, 2017). Each presynapse releases a single SV every 6 s for the first 33 s, resulting in a total of ∼60,000 SVs. Each SV is selected to have either a slow velocity (80% having 0.5 μm/s) or fast velocity (20% having 1.5 μm/s). Slow-velocity SVs are captured by presynapses with the same probability, regardless of the direction (0.75), while the retrograde capture probability of fast-velocity SVs varies depending on the simulation. Lastly, SVs are captured by the soma (x = 0) and are reflected at the farthest edge (x = 300). The advantage of this transient approach is that it allows us to distinguish flux rates and clearance rates for both fast motility (by measuring short-time-scale changes) and slow motility (by measuring long-time-scale changes) in the same simulation.

We used this model to estimate the flux rate of SVs into the soma as a function of retrograde capture probability. We measured the time each SV reaches the soma (left panel, Figure 6D) regardless of when/where it was released or how long it took to reach the soma (right panel, Figure 6D). We then measured the flux rate by integrating the number of SVs as a function of time (Figure 6E). All simulation conditions exhibited an initial fast flux rate, followed by a transition toward a slow flux rate; however, the simulations with lower retrograde capture probabilities (0.5 circles; 0.25 stars) than anterograde capture probabilities (0.75) had a significantly higher rate than those under the condition of equal retrograde/anterograde capture probability (0.75 squares). Furthermore, the lower-capture rate simulations resulted in an overall scale increase in the number of SVs that reached the soma that was equal to the fraction of fast-velocity SVs (20% of all SVs). Lastly, we quantified the fast and slow flux rates and showed that decreasing the retrograde capture probability alone is sufficient to increase the fast flux rate equivalent to the SVP anterograde flux rate previously measured (Watson et al., 2023). The net flux rate results support our hypothesis that a differential recycled SV capture probability leads to a net flux toward the soma.

We also used our model to measure the net SV density along the axon to determine the recycled SV clearance rate as a function of the distance from the soma. We first established that SVs with equal presynaptic capture probability (χ_Ret_ = χ_Ant_ = 0.75) do not travel far from their release location, as observed with the color-coded example tracks that tend to remain close to their location of release for at least 5 h after release (Figure 6G). As a consequence of this limited travel distance, the density of SVs per micron increases with the distance from the soma and remains elevated for the entire simulation (Figure 6H). In contrast, SVs with lower retrograde presynaptic capture probability (χ_Ret_ =0.25; χ_Ant_ = 0.75) quickly traverse the entire axon toward the soma (Figure 6I), resulting in a lower SV density as a function of the distance from the soma (Figure 6J), compared to equal SV capture probability (Figure 6H). These SV density results suggest that equal SV capture probability is insufficient to remove recycled SVs farther from the soma.

We separated the axon into three 100-μm-long regions relative to the soma (proximal, mid, and distal, Figure 6A) and quantified SV density as a function of time in each region as averages from the density measurements (Figures 6H, J). The resulting density as a function of time showed little difference in the proximal region for different retrograde capture probabilities (left panel, Figure 6K) but showed a significant overall difference in the number and rate of change in SV density for mid (middle panel, Figure 6K) and distal regions (right panel, Figure 6K). The total difference in SV density for mid and distal regions (∼20%) is equivalent to the fraction of SVs with fast velocity and having lower retrograde capture probability. We defined an SV clearance rate as the rate of change in SV density as a function of time (Figure 6L) and measured the clearance rate as linear fits to the fast and slow rates of change in the regional densities (Figure 6K). The fast clearance rate increased with decreasing retrograde capture rate and distance from the soma (top panel, Figure 6L), whereas the slow clearance rate decreased under all conditions as a function of increasing distance from the soma (bottom panel, Figure 6L). These SV clearance rate results support our hypothesis that a differential recycled SV capture probability supports the clearance of recycled SVs farther from the soma.

These combined flux rate and clearance rate model results show that the experimentally measured SV capture mechanics are essential for efficient SV recycling and are important for clearing SVs farther from the soma.

## 3 Discussion

Neurons must balance newly arriving protein-carrying vesicles with the removal of older protein-carrying SVs in order to maintain balance during homeostasis and presynaptic activity and provide support during plasticity. Extensive work has been carried out to understand how newly synthesized proteins are trafficked to presynaptic locations (Maeder et al., 2014; Watson et al., 2023). These studies have provided a valuable insight into the mechanics and rates of axonal protein trafficking. Furthermore, measurements of newly synthesized protein concentration at the local synaptic level have shown changes in the time scales due to activity dependence of turnover rates (Dörrbaum et al., 2018; Fornasiero et al., 2018; Heo et al., 2018; Dörrbaum et al., 2020; Jähne et al., 2021). However, this single-sided exploration leads to a fundamental problem—the process of removing older protein-carrying SVs has been left less understood. The removal of older protein-carrying SVs is especially important for neuronal plasticity and maintenance when the number and use of neurotransmitter carrying SVs are dynamically changing (Goda and Stevens, 1998). Thus, understanding the molecular mechanics of SV removal is essential to understanding how SV number and dynamics are balanced.

A central issue with understanding the mechanics of SV removal is whether they utilize both the actin and MT cytoskeletons or exclusively utilize the actin network during ISVE, as previously proposed. In this study, we show that SVs can utilize both the MT network (Figure 1), which has not been previously shown, and the actin network (Figure 3) during ISVE. However, the mechanics of SV travel during ISVE are observed to be independent of the direction of travel (Figure 2), which appears inconsistent with the results of previous studies (Hendricks et al., 2010; Hancock, 2014). Furthermore, we showed that SV pausing mechanics are influenced by both MT and actin motility (Figure 3), which suggests that SVs can utilize both cytoskeleton networks during ISVE transport. We then showed that SV capture rates by presynapses during ISVE are differentially mediated, with the retrograde capture rate being lower than the anterograde capture rate (Figure 4C). Furthermore, SV speed prior to reaching a presynapse distinguished whether it would be captured by the presynapse (Figure 4H).

These combined results led us to hypothesize that a two-state transport model distinguishes SV ISVE motility (Figure 5) and that bound myosin V is the mechanism that mediates whether SVs utilize actin or MT transport. SVs that leave a presynapse with bound myosin V travel slower, use actin-mediated transport, and are captured at higher fractions by neighboring presynapses. SVs that leave a presynapse without bound myosin V travel faster, utilize MT-mediated transport, and have lower retrograde capture fraction.

We then developed a theoretical framework and computational model to determine the cellular physiological consequences of the two-state transport model and differential capture fractions (Figure 6). We simulated ∼60,000 recycled SVs from 300 presynapses along an axonal lattice transiently released at a fixed rate and tracked their motility for ∼5 h. We then quantified the flux of SVs as they reached the soma and found that the fast-moving SVs with a lower retrograde capture probability (Figure 6F) were consistent with SVP flux rates leaving the soma (Watson et al., 2023). Furthermore, reducing the retrograde capture probability significantly decreased the flux rate, regardless of SV speed (Figure 6F). We then used this model to show that the SV clearance rate is mediated by the differential capture probability (Figures 6G–L). Importantly, reduced retrograde capture probability significantly affected the SV clearance rate as a function of the presynapse distance from the soma. Recycled SVs remained longer at presynapses farther from the soma.

Our transient mean-field SV release model approach used in the present study is important but limited due to its interpretation of physiological neuron turnover rates. Neurons regularly release SVs into the axon on a synapse-by-synapse basis. Furthermore, their release is dynamically regulated by activity and plasticity. However, we chose to use a transient release of SVs in order to distinguish the importance of retrograde capture probability on cellular-level flux rates. More robust and complete models should be developed in future studies that account for more complex presynaptic functions such as dynamically changing SV number, neuronal action potentials, exocytosis, and endocytosis. Our model provides an important initial framework.

One major limitation to our acute pharmacological approach is that it cannot distinguish the mechanics of how SVs lose myosin V during their life cycle. The agents MyoVin-1 and PBP inhibit myosin V, which resulted in lower capture fractions for SVs, supporting the role of myosin V. However, it is not clear from our approach if myosin V was still bound to SVs or if unbound myosin V can also re-bind to SVs. The dynamics of myosin V binding/unbinding are important to distinguish as they determine how SVs are dynamically selected to return to the soma. One possible mechanism for myosin V binding/unbinding would be the previously observed cross-linking between myosin V/synaptobrevin/synaptophysin (Prekeris and Terrian, 1997). A future study on this mechanism would directly image SV mobility while co-localizing synaptobrevin–synaptophysin and distinguish their role in the observed two-state model.

Another limitation to the significance of our results is that the dynamics observed using a hippocampal *in vitro* cell culture approach may not be directly translatable to other neuronal types or under physiological conditions. Hippocampal neurons *in vivo* have well characterized network connectivity pathways, with dynamically changing presynapse locations (Cappaert et al., 2015; Schinder et al., 2020). These cellular-level changes may influence the mechanics of how SVs transition in our two-state model or the capture/traverse fractions. The *in vitro* cell culture approach used in the present study did not control for either network connectivity or dynamically changing presynaptic density. Furthermore, network connectivity and synapse density change with neuron type and context (Sherwood et al., 2020; Serrano et al., 2022). Thus, future studies should explore SV capture/traverse dynamics using other neuronal types as well as *in vivo* slice labeling approaches to support and extend the results presented here.

One important finding and a missing gap in our present study is the relationship between SV capture/traverse fraction and presynapse size (Figure 4E). This result suggests that presynaptic structure may be a mediating factor with regard to whether SVs are captured, but presynapse size alone does not distinguish the structure. Presynapses have a complex spatially distributed actin cytoskeleton (Bingham et al., 2023), and it has also been established that the axonal MT network can dynamically change near presynaptic locations (Guedes-Dias et al., 2019). It is not clear whether there is a structure/size relationship, and our approach cannot distinguish this possibility. Future studies should explore this possibility further by imaging SV dynamics, followed by a more precise measurement of the presynaptic structure in the same neurons.

Another important missing gap in the present study is whether differential capture fractions are also dependent on MT-mediated motor motility. In the context of our two-state model, the lower capture/traverse fraction observed for fast-moving SVs was directly related to MT-driven transport (Figures 5B, F) and this capture/traverse difference directly impacted the flux rates at the soma (Figures 6E, F). Thus, how MT-mediated motility affects differential capture/traverse fractions is important as it would impact the flux rate of recycled SVs at the soma, and it would directly affect whether SV capture fractions can be dynamically modulated to accommodate changing demands (i.e., plasticity, presynaptic function, and SV recycling rates). Motor-mediated capture/traverse fractions have been explored in SVPs. SVPs have been established to target MT-end locations for both kinesin-driven motility (Guedes-Dias et al., 2019; Guedes-Dias and Holzbaur, 2019) and dynein-driven motility (Gramlich et al., 2021; Balseiro-Gómez et al., 2022). We showed that recycled SVs have a differential capture fraction dependent on direction (Figure 4K), but we did not distinguish whether this difference is mediated by molecular motor differences. Future studies should directly explore how MT-mediated transport affects capture/traverse fractions.

A major implication from our present study suggests that differential capture/traverse mechanics are essential in order to efficiently clear recycled SVs from presynapses farthest from the soma (Figures 6G–L). Independent of retrograde capture probability, SVs closest to the soma (proximal < 100 μm) are cleared at approximately the same rate, whereas high retrograde capture probability significantly affects SV mobility farthest from the soma (distal, >200 μm). Conversely, low retrograde capture probability results in the same SV clearance rate regardless of the distance from the soma (up to 300 μm, Figure 6L). This result suggests that a low retrograde capture bias is essential to maintain an efficient protein turnover with increasing presynaptic distance from the soma. Furthermore, the clearance rate results suggest that the efficiency of presynaptic function could quickly decrease with increasing distance from the soma due to a buildup of older SVs preventing newer SVPs from entering the region. Future studies could experimentally explore this possibility by distinguishing presynaptic function (release probability and protein-turnover rates) and distance from the soma.

Lastly, our results also support previous studies on the importance of captured SVs. ISVE has currently been established as an essential mechanism to maintain SV pool size and neurotransmitter release (Darcy et al., 2006; Park et al., 2012; Chen et al., 2021; Park et al., 2022). Our results show that a majority of recently recycled SVs are still captured by presynapses (Figures 4, 5), which suggests that neighboring presynapses regularly share SVs, as previously proposed. Our results expand on previous observations by also suggesting that shared SVs still have functionally bound proteins (inferred by bound myosin V) and thus can continue to support presynaptic function, as previously observed (Park et al., 2012; Park et al., 2022). Indeed, the loss of myosin V excludes non-functioning SVs from being captured, thus limiting efficient neurotransmitter release.

## 4 Materials and methods

### 4.1 Hippocampal cell cultures

Hippocampal cells were extracted from E19 Sprague–Dawley rat pups of mixed gender and dissociated via pipetting and incubation in papain medium (Gramlich et al., 2017). The cells were plated on previously prepared astrocytes on prepared glass coverslips in neuronal growth medium [84% Minimum Essential Medium (Thermo Fisher) with 9.6% Donor Equine Serum (HyClone), 2% 1 M glucose in MEM (Thermo Fisher), 0.5% penicillin/streptomycin (Thermo Fisher), 1% N_2_ supplement (Thermo Fisher), 1% sodium pyruvate (v), and 2% 1 M HEPES, by volume]. The plated cells were then placed in an incubator until imaging. After 24–48 h, the neuronal growth medium was replaced by enriched neurobasal medium [96% Neurobasal-A Medium (Thermo Fisher), 2.5% B-27 supplement (Thermo Fisher), 0.3% GlutaMAX-1 (Thermo Fisher), and 1.2% penicillin/streptomycin (Thermo Fisher), by volume]. After 7 DIV, an additional 0.5 ml of enriched neurobasal medium was added.

### 4.2 Fluorescence microscopy and imaging protocols

At 14–20 DIV, cell culture dishes were transferred onto a custom-built microscope and exposed to imaging medium (125 mM NaCl, 2.55 mM KCl, 10 mM HEPES, 15 mM glucose, 5 µM CNQX, 2 mM CaCl_2_, and 4 mM MgCl_2_, pH = 7.25). The cells were imaged using a Nikon Eclipse Ti2 microscope base (Nikon) equipped with a ×100 oil immersion objective. Images were recorded using a Hamamatsu Flash v4 CMOS camera (Hamamatsu), with a pixel resolution of 65 nm/pixel. The entire microscope was enclosed in an incubator (Oko Labs) maintained at 37°C. The cells were exposed to a backlit LED light source using either a GFP (Nikon) or FITC (Nikon) cube for SGC5 or EB3-RFP imaging, respectively. Samples were regularly perfused with imaging medium during the experiment. SGC5 imaging was obtained with an 80 ms frame exposure and 10 Hz frame rate. EB3 imaging was obtained with 80 ms exposure and a 1 Hz frame rate.

### 4.3 Cell culture stimulation and SGC5 dye loading

Cells were stimulated to induce Ca^2+^ influx using a pulse generator (B&K Precision) connected to platinum electrodes submerged in imaging medium 1 mm above the cells spaced 1 cm apart. Each pulse occurred for 1 ms, with a depolarizing voltage of +75 mV at the cells. Single-vesicle loading involved a paired pulse 200 ms apart, while cells were exposed to imaging medium containing 10 μM SGC5 (Invitrogen), followed by a 30-s delay for vesicle exocytosis. The cells were then washed for 4 min to remove excess dye and were imaged. Bulk SGC5 loading involved 200 pulses at 50 Hz, while cells were exposed to medium containing 10 μM SGC5, followed by a 1-min delay. The cells were then washed to remove excess dye and were imaged. During bulk imaging, the cells were stimulated with 900 pulses at 30 Hz while continuously imaged.

For any sample to be included in our analysis, it must go through three rounds of stimulation (single-SV SGC5 loading, bulk SV SGC5 loading, and bulk SGC5 unloading), without disruption of the plasma membrane distinguished as SGC5 loaded in lipid bubbles. SGC5 lipid bubbles are defined as spherical regions of SGC5 intensity >2 μm in radius. If a synaptic vesicle track exhibits single-SV SGC5 but overlaps with SGC5-loaded lipid bubbles during bulk loading/unloading, then it is excluded from the analysis. This restriction prevents bias from neurons that have compromised plasma membrane integrity in the results.

### 4.4 Lentiviral transfection

At 3 DIV, the cell-enriched neurobasal medium was removed and replaced with an EB3–RFP lentiviral vector (LentiBrite EB3-RFP Lentiviral Biosensor, MilliporeSigma) at a concentration of 2.75 × 10^6^ IFU/ml for a multiplicity of infection of 27.5. Cells were returned to incubator for 1 day, then transfection media was removed and replaced with 1 ml of enriched neurobasal medium. After 7 days, an additional 0.5 ml of enriched neurobasal medium was added. Incubation was continued as normal until 14–20 DIV, and then the cells were imaged.

### 4.5 Pharmacology

For nocodazole condition experiments, cells were exposed to 1–2 nM nocodazole (Sigma, diluted in DMSO) in imaging medium and incubated for 10 min prior to fluorescence dye loading. This concentration was chosen as it was previously shown to stabilize microtubules and reduce microtubule dynamics (Vasquez et al., 1997). For myosin V-inhibited experiments, cells were exposed to either a concentration of 30 μM MyoVin-1 (Sigma, diluted in DMSO) in imaging medium for 10 min or 5 nM PBP (Fisher Scientific, diluted in DMSO) for 5 min in imaging medium prior to fluorescence dye loading. These (MyoVin-1/PBP) concentrations were chosen as they were previously shown to inhibit myosin V motility without targeting other myosin-family motors (Bond et al., 2012). For Arp2/3-inhibited experiments, cells exposed to concentrations of 68 μM of CK-666 (MilliporeSigma, diluted in DMSO) in imaging medium were incubated for 10 min prior to fluorescence dye loading. The CK-666 concentration was based on MilliporeSigma-recommended guidelines and published IC_50_ tables. For depolymerized actin experiments, samples were incubated with 30 μM latrunculin-A (Lat-A, Fisher Scientific, diluted in ethanol) in imaging medium for 30 min prior to fluorescence dye loading. This Lat-A concentration was chosen as it destabilizes the actin network without resulting in cell death.

## 5 Image and data analyses

### 5.1 Kymograph analysis

Long-range (>1 µm) vesicle tracks were identified from raw data based on the total distance traveled during experimental imaging along identified axonal processes in bulk SGC5 loading (Figure 1B). Kymographs were then created using ImageJ using the standard plugin KymographBuilder (Figure 1D). Directed SV transport time points were identified as the changing intensity along both the (x,t)-axes of the kymograph, whereas pauses were identified as locations of constant intensity at the same x-location on the kymograph, shown as vertical lines on the kymograph (Figure 1D).

SV speed was measured from kymographs by calculating the slope of a line during directed motion using intervals of 1 μm to identify changes in SV speed during travel. The slope was then converted to μm/s using our nm/pixel resolution and recording times. Pause times were measured following our previously utilized protocol (Gramlich et al., 2021). In brief, a line was drawn along the SV trajectory before the pause and another line along the SV trajectory after the pause, with the vertical distance between these two lines reported as the pause time. Given the finite width of the vesicle in the image, the smallest pause time (shift between diagonal lines) was two frames, or 0.2 s. The distance between two pauses was measured from the start of one pause to the start of the next pause (Figure 1D).

### 5.2 Single-vesicle tracking and correlation analysis and pause identification

SVs were tracked using established MATLAB algorithms that fit each SGC5 peak in a frame using two-dimensional Gaussian fitting and linking fits in different frames together as a single track (Jaqaman et al., 2008; Gramlich and Klyachko, 2017). Individual track data (x,y, amplitude) were then analyzed using our previously established correlation algorithm that identifies periods of motor-driven (fast), intermediate (diffusive), or paused (pause) times (Gramlich and Klyachko, 2017). Track data and the correlated motion condition were then combined and output as a single file.

Axonal tracks were identified as traveling further than 1 μm during observation. Axonal pauses were identified as either not co-localizing within 500 nm of identified bulk SGC5 loading/unloading locations or during periods of dense point regions that span a circular diameter of 500 nm in the track data (presynapse, Figure 3B). Aggregate speeds, time in runs, time in pauses, and run-lengths were combined on an individual run basis (Figure 2). Aggregate pausing analysis was performed using the following protocol: (i) bouts of fast motion, followed by bouts of pausing, were identified (Figure 3B); (ii) the frame initiating a pause was defined as t = 0 for each pause identified; (iii) the SV correlation and displacement metrics (described below) were then averaged on a frame-by-frame basis relative to t = 0 (where –t is the time before the pause and +t is time during the pause); and (iv) average results, along with SEM, were then reported as a function of time relative to the onset of pausing (Figures 3E–J).

We note that a small fraction of SV tracks exhibited reversals after pausing (e.g., five reversals in all control tracks <7%). Pauses followed by reversals were not included in the pause time comparison to avoid ambiguity and in case a more complicated motor-driven process is responsible for reversal behavior.

### 5.3 Single-vesicle axonal geometry identification and displacement analysis

Identified uTrack data were filtered using a three-frame moving average (Gramlich and Klyachko, 2017). The x and y data points were then run through an in-laboratory-built fitting algorithm that involved the following steps: (i) identify regions of dense track points (>16 points per region) using a moving window of 100 nm × 100 nm that scanned across the data and excluded them from axonal fitting; (ii) fit regions of low-density points to either a straight line or semi-circle determined by least-squares residual fitting to 16 data point fit windows and a maximum circularity ratio at any given point in the fit (0.089); and (iii) draw a single contiguous axonal line from the beginning of the track data to the end connecting identified regions of lines and semi-circles, with straight lines drawn across regions of dense points as the end point minus the start point (black line, Figure 3B).

The motion of each track data point was then calculated relative to the axon fit line. At each point, a track displacement vector was calculated, as previously described (Gramlich and Klyachko, 2017). The displacement vector was then split into two vectors that were perpendicular or parallel to the axon fit line. The absolute displacement of the track point from the axon fit line was then calculated. The amplitude and angle of each vector were relative to the axon fit line at each data point. The axon fit data were then combined with the correlation analysis results for each track point so that the type of motion and the direction of motion relative to the axon fit line were reported for each track data point. These combined metrics were then aggregated to determine the mean track displacement (Figure 3).

### 5.4 Measuring EB3 location and SV co-localization

To measure the ends of microtubules, we utilized fluorescent markers attached to the microtubule-associated protein end-binding protein 3 (EB3–RFP) (Figure 1C). EB3 images were collected using a single-SV SGC5 loading protocol but just prior to SV video collection. Using the characteristic profile of an EB3 point and the measured velocities of EB3 in our samples, we projected the EB3 point motion in time to determine where an SV would intersect with an EB3 point during the SV movie. EB3 location was measured as the leading peak of a smoothed EB3 profile. This location was then reported on the SV kymograph with an error of ±0.5 µm in width to account for EB3 punctum size and possible variations in speed or timing. SV motion (motor-driven or pausing) was then correlated at the co-localization point of the EB3 puncta on the kymograph. An extra exclusion criterion was used to count an interaction between the EB3 puncta and SV motion, where an SV that paused at a location prior to the projected presence of an EB3 point on the SV kymograph was not counted. We further measured pause and traverse locations for points not associated with projected EB3 locations and reported the pause traverse fraction as a function of location relative to the projected EB3 location (Figure 1E).

#### 5.4.1 Spacing correlation

SV-pause spacing and EB3 punctum distance were correlated in the same local axonal region where both were measured to occur. First, spacing between SV pauses was measured as mentioned previously, where at least one or more pauses were observed to occur. Then, spacing between EB3 puncta was measured using a line drawn along the same axonal process between two EB3 puncta, with a restriction that EB3 puncta were counted less than ∼10 µm from SV-pausing. If there were three or more pauses in a kymograph or three or more EB3 puncta on the same axon, each spacing was measured, and then all measurements were averaged. Multiple SV-pause spacings were then averaged in 0.5-µm bins with SEM of the average results reported (Figure 1F).

#### 5.4.2 Presynapse identification

Presynapses were identified after single-SV movies were collected. First, the single-SV protocol was used to identify SV motion. Then, the aforementioned bulk SGC5 loading protocol was followed. Bulk movies were then collected, and locations with decreasing SGC5 intensity during stimulation were identified. Presynapses were then identified as either having an average SGC5 intensity ∼10 times the measured single-SV intensity and/or exhibiting decreasing SGC5 intensity during stimulation.

## 6 Computational modeling approaches

### 6.1 SV ISVE pausing mechanics

We computationally simulated SV axonal pausing during ISVE between presynapses using our previously established computational approaches in Python (Gramlich et al., 2021). Each simulation followed the same protocol, as explained in the following paragraphs.

First, SVs travel with fixed fast-directed speed (determined from experimental values, Figure 3) and a maximum angle for each time step drawn from a random normal distribution (mean = 15°) for 40 time steps.

Second, at 40 time steps, SVs begin to slow at a rate of ∼10 nm/time step and increase their maximum angle (mean of 25°).

Third, at 50 time steps, an axonal axis is calculated as a line that fits the first 50 frames. This line is extrapolated forward in space and represents the axonal axis of the simulated track.

Fourth, the SV initiates a pause relative to the axonal axis line determined. The SV has a maximum displacement of 15 nm/time step. The direction of displacement is chosen randomly from a predetermined distribution that is (i) biased away from the axonal axis line if the SV is less than 60 nm from the line and (ii) biased toward the axonal axis line when it is farther than 250 nm away. This approach keeps SVs bound within a region of displacement relative to the axonal axis line.

Fifth, the SV pauses for a minimum of six time steps, equivalent to the minimum number of frames used in the correlation analysis (Figures 2, 3). The SV remains paused for a random number of time steps drawn from an exponential distribution with means taken from experimentally determined values (Figure 3C).

Sixth, the SV resumes fast-directed transport after the pause ends until a time of 700 time steps (equivalent to 700 frames imaged experimentally).

Seventh, the resulting (x, y, t) track data are run through the correlation and axon axis algorithms used for experimental data. The quantified parallel, perpendicular, displacement, and pause-time results are then compared to experimentally observed results (Figure 3).

### 6.2 Axonal SV trafficking and differential capture mechanics

We computationally simulated SV trafficking along axons with a regular distribution of presynapses using our previously established computational approaches in Python (Glomb et al., 2023). Each simulation followed the same protocol as described in the following paragraphs (Computational code can be found on github: 10.5281/zenodo.10048388).

First, we defined an axon as a one-dimensional lattice of fixed lattice sites (equal to 100 nm) and simulation time steps equal to 0.2 s each. The lattice had a defined length of 310 lattice sites (310 μm). The soma was defined at the lattice site x = 0, and the axon growth cone was defined as a reflecting boundary where all SVs reversed direction and continued traveling.

Second, we defined locations at fixed distances along the axon (30 lattice sites, 3 microns) as presynapse locations. Each simulated SV originated from one of these locations. Any SV that traverses a designated presynapse location has a fixed probability of capture, with anterograde capture for all simulations remaining constant (0.75) and retrograde capture having one of three values (0.25, 0.5, and 0.75) that is predefined at the beginning of the simulation. SVs that are captured by a presynapse pause for a random amount of time drawn from an exponential distribution with a mean value of 300 time steps (60 s), which is approximately equal to the inverse of the exit rate.

Third, we simulated individual SV tracks originating from a presynapse site for up to 20,000 time steps (∼5.5 h modeled simulation time), where each track was simulated independent of the other tracks. We simulated 100 tracks per presynapse, with 300 presynapses for a total of 3,000 unique tracks. If an SV reaches the soma, then the simulation is ended. If the SV reaches the axon end (x = 310), it reverses direction.

Fourth, we then simulated an entire axon of presynapses and SVs by using a bootstrapping approach to randomly draw a single SV from each presynapse at fixed times during simulation. We randomly chose a single SV from each presynapse at a fixed time interval (every 30 time steps). The SV track was then added to the aggregate axonal lattice independent of the other SVs. SVs were added to the simulation for 200 time steps, resulting in a total of 60,000 SVs per simulation.

Fifth, the time taken by each SV to reach the soma was counted in an array and used for flux analysis. The number of SVs in each lattice site was counted and stored for density and clearance rate calculations.

## 7 Statistical analyses

Statistical comparisons that involved fraction analysis (pause/traverse, Figures 1E, 4F, G) were performed using a comparison of two proportions applying a chi-squared distribution analysis to each condition and comparing the resulting distributions using MATLAB (Laurie, 2023). Comparisons of repeated measures of SV mechanics within a group (i.e., before pause compared to during pause; Figures 3C–H, 4H–J) were performed using a repeated-measures *t*-test. Comparisons of repeated measures of SV mechanics between groups (i.e., CT, Myo-1, CK-666, and Noc) were performed using Mann–Whitney U-tests.

## Data Availability

The raw data supporting the conclusion of this article will be made available by the authors, without undue reservation.
