# Supplementary figures and images for: Recently recycled synaptic vesicles use multi-cytoskeletal transport and differential presynaptic capture probability to establish a retrograde net flux during ISVE in central neurons
Source: Front Cell Dev Biol. 2023 Nov 6;11:1286915. doi: 10.3389/fcell.2023.1286915 (PMC10657820; doi:10.3389/fcell.2023.1286915)

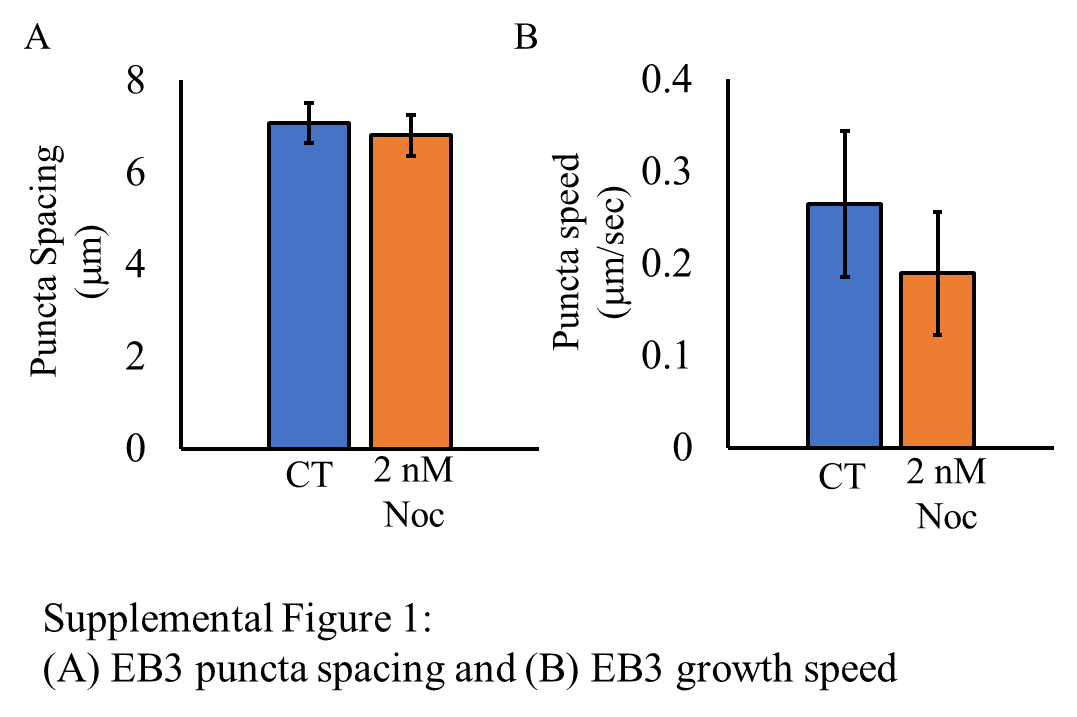

Supplement: Supplementary file 1 [file Image1.tiff]

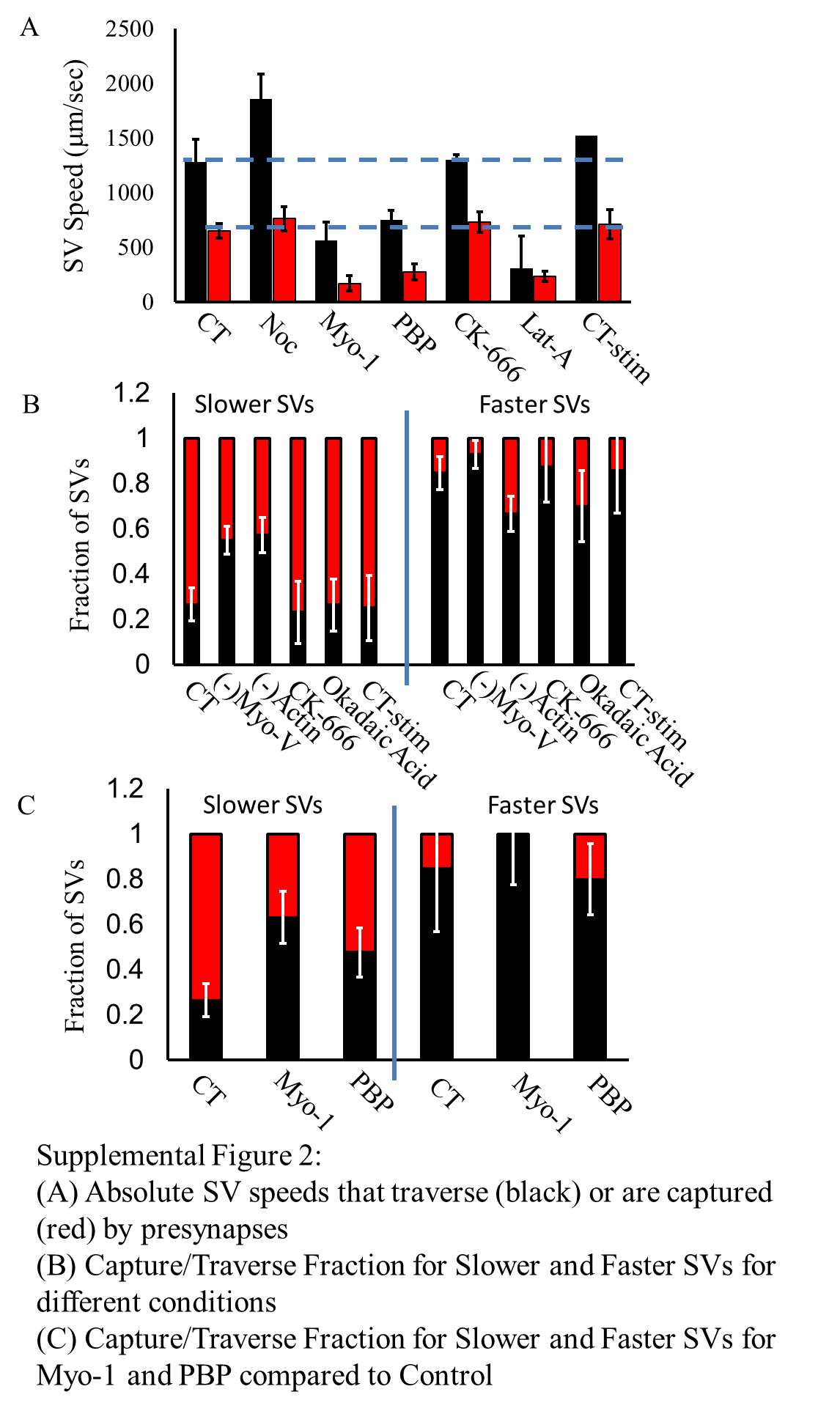

Supplement: Supplementary file 3 [file Image2.tiff]
